# Neuronal DAF-16-to-intestinal DAF-16 communication underlies organismal lifespan extension in *C. elegans*

**DOI:** 10.1016/j.isci.2021.102706

**Published:** 2021-06-10

**Authors:** Masaharu Uno, Yuri Tani, Masanori Nono, Emiko Okabe, Saya Kishimoto, Chika Takahashi, Ryoji Abe, Takuya Kurihara, Eisuke Nishida

**Affiliations:** 1 RIKEN Center for Biosystems Dynamics Research (BDR), Minatojima-minamimachi, Chuo-ku, Kobe 650-0047, Japan; 2 Department of Cell and Developmental Biology, Graduate School of Biostudies, Kyoto University, Sakyo-ku, Kyoto 606-8502, Japan

**Keywords:** Biological sciences, Genetics, Cell biology

## Abstract

Previous studies have revealed the importance of inter-tissue communications for lifespan regulation. However, the inter-tissue network responsible for lifespan regulation is not well understood, even in a simple organism *Caenorhabditis elegans*. To understand the mechanisms underlying systemic lifespan regulation, we focused on lifespan regulation by the insulin/insulin-like growth factor-1 signaling (IIS) pathway; IIS reduction activates the DAF-16/FOXO transcription factor, which results in lifespan extension. Our tissue-specific knockdown and knockout analyses demonstrated that IIS reduction in neurons and the intestine markedly extended lifespan. DAF-16 activation in neurons resulted in DAF-16 activation in the intestine and vice versa. Our dual gene manipulation method revealed that intestinal and neuronal DAF-16 mediate longevity induced by *daf-2* knockout in neurons and the intestine, respectively. In addition, the systemic regulation of intestinal DAF-16 required the IIS pathway in intestinal and neurons. Collectively, these results highlight the importance of the neuronal DAF-16-to-intestinal DAF-16 communication for organismal lifespan regulation.

## Highlights

• Neurons and the intestine are important in the regulation of lifespan
• Neuronal *daf-2* KO activates not only neuronal DAF-16 but also intestinal DAF-16
• Intestinal *daf-2* KO activates not only intestinal DAF-16 but also neuronal DAF-16
• DAF-16-to-DAF-16 communication between neurons and the intestine regulates lifespan

## Graphical abstract

**Figure:**
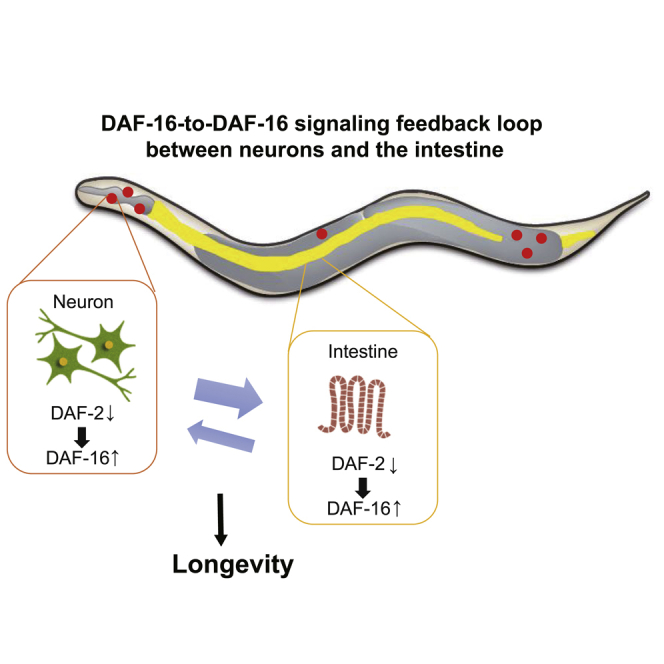

## Introduction

As organisms developed multicellularity, those with specialized cells, tissues, and organs emerged during evolution. To achieve systemic regulation in multicellular organisms with specialized tissues, inter-tissue communication systems evolved. The central nervous system influences many tissue behaviors via hormones or neurons. Recent studies have suggested that tissues other than those in the nervous system can also regulate other tissues involved in many biological processes (Moskalev, 2015). Thus, the examination of inter-tissue communications is the key to understanding the behavior of multicellular organisms. Consistently, recent studies have shown that aging is also regulated in a systemic fashion (Taylor and Dillin, 2013; Leiser et al., 2015; Burkewitz et al., 2015; Durieux et al., 2011), although the inter-tissue communications underlying lifespan regulation remain largely unknown.

After the identification of the insulin/insulin-like growth factor-1 (IGF-1) signaling (IIS) pathway as a lifespan regulating pathway in *C*. *elegans* (Kenyon et al., 1993), the association between the IIS pathway reduction and longevity has been established in diverse model organisms using genetic interventions (Kenyon, 2010). In *C*. *elegans*, reduced IIS pathway caused by mutations of *daf-2*, the *C*. *elegans* homolog of the insulin/IGF-1 receptor (Kimura et al., 1997), or by those of *age-1*, the *C*. *elegans* homolog of phosphatidylinositol 3-kinase (Friedman and Johnson, 1988a; 1988b), extends the lifespan, which is dependent on DAF-16, the *C*. *elegans* homolog of the forkhead box FoxO transcription factor (Ogg et al., 1997; Lin et al., 1997). The tissue requirements of IIS components have previously been analyzed only by tissue-specific restoration experiments; restoration experiments for DAF-2 and DAF-16 in *daf-2* and *daf-16*; *daf-2* mutants, respectively, revealed that DAF-2 in neurons and DAF-16 in the intestine play an important role in lifespan regulation (Wolkow et al., 2000; Iser and Wolkow, 2007; Libina et al., 2003). However, systemic lifespan regulation in *C*. *elegans* via the IIS pathway is not fully understood.

In this study, we explored systemic lifespan regulation by the IIS pathway. Our tissue-specific knockdown and knockout of *daf-2* and/or *daf-16* confirmed that the IIS pathway in neurons and the intestine has an important role in lifespan regulation. Our experiments, in which the activities of DAF-2 and DAF-16 are manipulated in neurons and the intestine, have clearly shown that the longevity induced by *daf-2* knockout in neurons requires DAF-16 function in the intestine, and the longevity induced by *daf-2* knockout in the intestine requires DAF-16 function in neurons. Thus, our study demonstrates that the neuron-to-intestine communication via the IIS pathway regulates organismal lifespan.

## Results

### IIS pathway in neurons and the intestine has a role in the regulation of the lifespan

**Figure 1:**
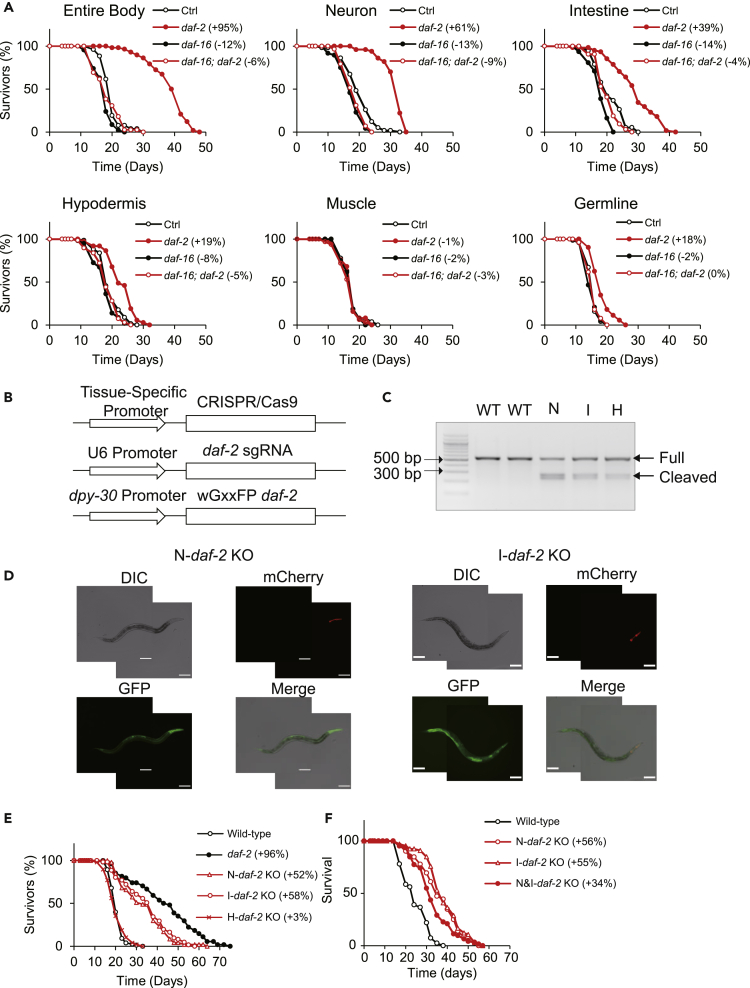
The IIS pathway in neurons and the intestine is involved in the regulation of the lifespan (A) Survival curves of the worms treated with *daf-2* and/or *daf-16* RNAi in the entire body (wild-type N2, upper left), neurons (TU3401, upper middle), the intestine (VP303, upper right), the hypodermis (NR222, lower left), the muscle (NR350, lower middle), or germline cells (DCL569, lower right). Representative data from two or three independent experiments are shown. (B) Scheme showing the method used to obtain tissue-specific *daf-2* knockout worms. CRISPR/Cas9 was driven by a tissue-specific promoter (neurons: P*rgef-1*, intestine: P*gly-19*, and hypodermis: P*dpy-5*), and *daf-2* sgRNA utilized the U6 promoter. To determine the tissues in which CRISPR/Cas9 was successful, wGxxFP driven by a ubiquitous promoter (P*dpy-30*) was introduced (see text for detail). (C) Nuclease-driven mutations were detected by T7E1 assay. Representative DNA gel image of the T7E1 assay shows *daf-2* PCR products amplified from genomic DNA from N2 worms (WT) and worms with extrachromosomal arrays for *daf-2* knockout in neurons (N), the intestine (I), or the hypodermis (H). ‘Full’ and ‘Cleaved’ indicate the expected positions of DNA bands of *daf-2* PCR products and those of DNA bands cleaved by mismatch-sensitive T7E1, respectively. (D) Representative expression patterns of wGFP, reconstituted from wGxxFP, as observed in neuronal *daf-2* knockout (N-*daf-2*) or intestinal *daf-2* knockout (I-*daf-2*) KO transgenic worms. Scale bar indicates 100 μm. (E) Survival curves of worms with the knockout of *daf-2* in neurons (N-*daf-2*), the intestine (I-*daf-2*), or the hypodermis (H-*daf-2*). Representative data from three independent experiments are shown. (F) Survival curves of worms with the knockout of *daf-2* in neurons (N-*daf-2*), the intestine (I-*daf-2*), or both neurons and the intestine (N&I-*daf-2*). Representative data from three independent experiments are shown. Statistics are presented in Tables S1, S2, and S5.

Tissue-specific restoration experiments have previously been performed to analyze the tissue requirements of IIS pathway components. Here, we first verified the tissues involved in IIS signaling-mediated lifespan regulation by tissue-specific gene knockdown experiments. We utilized strains that are able to process RNAi efficiently only in particular tissues but not in other tissues (neuron: TU3401 [Calixto et al., 2010] intestine: VP303 [Espelt et al., 2005]; hypodermis: NR222 [Qadota et al., 2007]; muscle: NR350 [Qadota et al., 2007]; germline cells: DCL569 [Zou et al., 2019]). Using these strains, we conducted tissue-specific *daf-2* and/or *daf-16* knockdown experiments (Kamath et al., 2003). As reported (Kenyon et al., 1993), *daf-2* knockdown doubled the lifespan of wild-type N2 organisms, and this lifespan extension was abolished by simultaneous *daf-16*; *daf-2* knockdown (Figure 1A and Table S1). Our results showed that *daf-2* knockdown in neurons, the intestine, the hypodermis, and germline cells significantly increased lifespan, which is consistent with the findings of previous studies (Wolkow et al., 2000; Iser and Wolkow, 2007; Libina et al., 2003; Kwon et al., 2010) that showed the reduction of the IIS pathway in neurons, the intestine, and the hypodermis extended lifespan. In contrast, muscle-specific *daf-2* knockdown did not increase lifespan, which is also consistent with the findings of previous studies (Wolkow et al., 2000; Libina et al., 2003). However, the possibility cannot be ruled out that the unextended lifespan in muscle-specific RNAi animals may result from the inefficiency of the RNAi treatment. When we simultaneously knocked down *daf-16* and *daf-2* in the same tissue, the longevity increase induced by tissue-specific *daf-2* knockdown in neurons, the intestine, the hypodermis, and germline cells was completely suppressed (Figure 1A and Table S1). In addition to lifespan regulation, *daf-2* knockdown in neurons and the intestine, also significantly increased resistance to oxidative stress, and this increase in stress resistance was suppressed by *daf-16* knockdown in the same tissue (Figure S1A and Table S2). These results suggest that the reduction of the IIS pathway in neurons and the intestine increases lifespan as well as oxidative stress resistance, and these increases require DAF-16 in each tissue.

We focused on the regulation of lifespan by the IIS pathway in somatic tissues (neurons, the intestine, and the hypodermis). To identify the somatic tissues responsible for lifespan regulation, we performed knockout experiments. According to the method used in a previous study (Shen et al., 2014), we knocked out *daf-2* in neurons, the intestine, or the hypodermis by using CRISPR/Cas9 with the *rgef-1*, *gly-19*, or *dpy-5* promoter, respectively (Figure 1B). A T7E1 assay confirmed that Cas9 guided by *daf-2* sgRNA could digest the target region (Figure 1C). To visualize the tissues in which *daf-2* knockout was performed, we introduced *daf-2* sgRNA and Cas9 with a *dpy-30* promoter (all somatic tissue) driven by wGxxFP, which is reconstituted into GFP after homology dependent repair (HDR) caused by *daf-2* sgRNA-guided Cas9-induced digestion (Mashiko et al., 2013) (Figure 1B). When we introduced Cas9 into neurons, the intestine, and the hypodermis, we detected GFP fluorescence in neurons, the intestine, and the hypodermis, respectively (Figures 1D and S1B), suggesting that our tissue-specific knockout experiment worked properly.

Tissue-specific *daf-2* knockout in neurons, the intestine, and the hypodermis resulted in the smaller body size compared to the wild-type animals (Figure S1D). Also, *daf-2* knockout in neurons significantly increased the dauer formation ratio at 25°C (Figure S1E and Table S3), which is consistent with the previous observation (Libina et al., 2003). Tissue-specific *daf-2* knockout in neurons and the intestine significantly increased lifespan, while the effect on lifespan of *daf-2* knockout in hypodermis varied between individual trials: one trial found little effect on longevity of statistical significance, while the other two trials found no effect on lifespan (Figure 1E and Table S2). Consistently, *daf-2* knockout in neurons and the intestine, but not in hypodermis, increased reproductive span similarly to how the *daf-2* mutation increased reproductive span (Figure S1F and Table S4). Interestingly, simultaneous *daf-2* knockout in both neurons and the intestine did not further extend the lifespan as was observed for either *daf-2* knockout in neurons or the intestine (one trial showed longer lifespan, while the other two trials showed shorter lifespan: Figures 1F and S1C and Table S5). To evaluate the toxicity of Cas9 overexpression, we expressed Cas9 in all somatic tissues (driven by the *eft-3* promoter) with empty or gfp sgRNA. Our measurements showed that the overexpression of Cas9 with an empty or gfp sgRNA did not change the overall lifespan or reproductive span (Figures S1G, S1H and Tables S6 and S7), which thus demonstrates the lack of toxicity caused by Cas9 overexpression. Overall, the reduced IIS pathway in neurons and the intestine is able to extend lifespan at least partly through a shared mechanism.

As *daf-2* knockout in neurons significantly increased lifespan, we asked whether neuropeptide acted downstream of neuronal DAF-2 to control lifespan. To test this, we utilized the *unc-31 (e928)* mutant strain (which is defective in the release of dense core vesicles containing neuropeptides) (Speese et al., 2007). Thus, we knocked out *daf-2* in neurons of the *unc-31* mutant strain. Our lifespan measurements showed that the *unc-31* mutation increased lifespan of wild-type animals as reported (Ailion et al., 1999) and significantly increased lifespan of neuronal *daf-2* knockout animals (Figure S2A and Table S5). The statistical analysis suggests that there could be a functional interaction between neuronal DAF-2 and neuropeptide release (Table S5): deficiency in neuronal DAF-2 could reduce neuropeptide release, or deficiency in neuropeptide release could inhibit neuronal DAF-2 function.

### DAF-16 activation in neurons led to DAF-16 activation in the intestine, and vice versa

**Figure 2:**
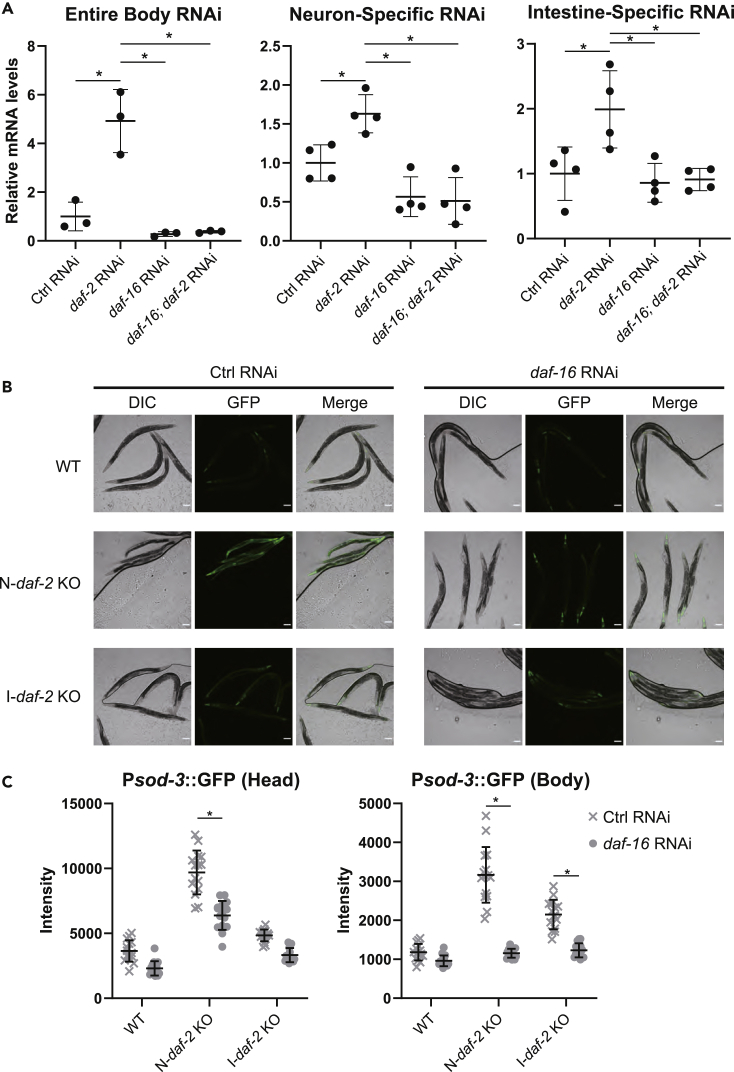
Deficiency of *daf-2* in neurons or the intestine activates SOD-3 expression in the intestine or neurons, respectively (A) The quantitative RT-PCR (qRT-PCR) of *sod-3* expression in worms treated with *daf-2* and/or *daf-16* RNAi in the entire body (wild-type N2, left), neurons (TU3401, middle), or the intestine (VP303, right). Statistical significance was calculated by one-way ANOVA with a post hoc Tukey's test. ∗p < 0.05. Error bars represent the mean ± s.d. of three or four independent experiments. (B) Representative images showing the expression pattern of GFP driven by the promoter of *sod-3*, a well-known DAF-16 target, in wildtype (upper) and transgenic worms with knockout of the *daf-2* gene in neurons (N-*daf-2* KO, middle) or the intestine (I-*daf-2* KO, lower). Each strain was treated with control (left) or *daf-16* (right) RNAi. Scale bar indicates 100 μm. (C) Quantification of GFP fluorescence intensity of head (left) or body (right) region of animals. Representative data from two independent experiments are shown. Data represent mean ± s.d. from 15 individual animals. Statistical significance was calculated by two-way ANOVA with a post hoc Sidak's test. ∗p < 0.05.

**Figure 3:**
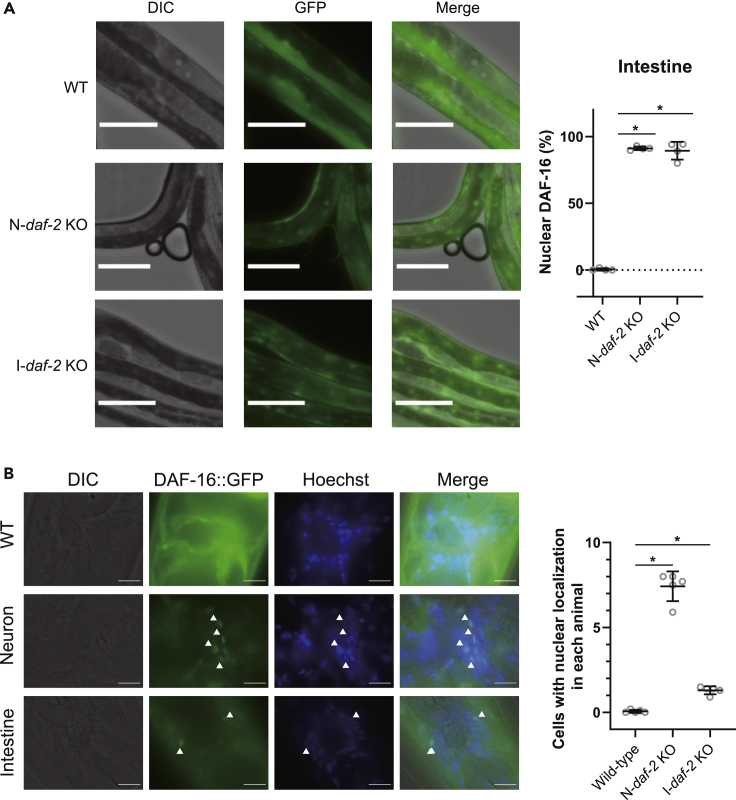
Deficiency of *daf-2* in neurons or the intestine activates DAF-16 in the intestine or neurons, respectively (A) Localization pattern of DAF-16::GFP in the body region of wildtype (left-upper) and that of transgenic worms with knockout of the *daf-2* gene in neurons (N-*daf-2* KO, left-middle) or the intestine (I-*daf-2* KO, left-lower). Scale bar indicates 100 μm. Ratio of the animals with DAF-16::GFP nuclear accumulation (right). Data represent mean ± s.d. from 4 independent experiments. Statistical significance was calculated by one-way ANOVA with a post hoc Tukey's test. ∗p < 0.05. (B) Expression patterns of DAF-16::GFP in the head region of wildtype (upper) and transgenic worms with knockout of the *daf-2* gene in neurons (N-*daf-2* KO, middle) or the intestine (I-*daf-2* KO, lower) (left). Scale bar indicates 10 μm. Scatterplot indicate the mean number of cells with nuclear localization in each animal from 5 independent experiments (right). Data represent mean ± s.d. from 5 independent experiments. Statistical significance was calculated by one-way ANOVA with a post hoc Dunnett's test. ∗p < 0.05. Statistics are presented in Tables S8 and S9.

The longevity increase induced by *daf-2* knockout in neurons or the intestine was similar in our experiment; however, previous tissue-specific DAF-16 restoration experiments in the *daf-2*; *daf-16* double mutant showed that the restoration of DAF-16 in the intestine increased lifespan more efficiently than in neurons (Libina et al., 2003). This apparent difference (Libina et al., 2003) may imply that the reduction of the IIS pathway in neurons requires the presence of DAF-16 in other tissues. In other words, inter-tissue DAF-16-to-DAF-16 signaling may contribute to maximizing the anti-aging effects induced by the neuronal IIS pathway. Thus, we examined whether *daf-2* deficiency in neurons affects DAF-16 activity in the intestine and vice versa. First, we quantified the mRNA expression levels of *sod-3*, a well-known target of DAF-16 (Honda and Honda, 1999; Furuyama et al., 2000), and found that *daf-2* knockdown in the intestine or neurons significantly increased *sod-3* mRNA levels in a DAF-16-dependent manner (Figure 2A). Then, we examined the expression of GFP driven by the *sod-3* promoter under conditions in which *daf-2* was knocked out in neurons or the intestine. Because most of the neurons localize in the head region and the intestine is a main organ that expresses GFP in the body region, we measured the expression of GFP in the head and body regions. Consistent with the qPCR analyses, both neuronal *daf-2* knockout and intestinal *daf-2* knockout clearly increased the expression of GFP (Figure S2B). While neuronal *daf-2* knockout increased the GFP expression in the head region markedly, intestinal *daf-2* knockout increased it modestly (Figure S2C, left). Both neuronal *daf-2* knockout and intestinal *daf-2* knockout clearly increased the GFP expression in the body region (Figure S2C, right). Treatment with *daf-16* RNAi suppressed GFP increase in the intestine induced by *daf-2* knockout in the head or body region (Figures 2B and 2C), suggesting that the reduction of the IIS pathway in neurons or the intestine activates DAF-16 in the intestine. Also, *daf-16* RNAi suppressed GFP increase in neurons induced by *daf-2* knockout in the head region (Figure 2C, left). We then examined whether neuronal *daf-2* knockout activates intestinal DAF-16 and vice versa. To test this hypothesis, we knocked out neuronal or intestinal *daf-2* in transgenic animals expressing DAF-16::GFP (TJ356). Neuronal *daf-2* knockout clearly increased the nuclear accumulation of DAF-16::GFP in the intestine as well as in neurons (Figures 3A, 3B, and S2D and Table S8). Intestinal *daf-2* knockout modestly but clearly increased the nuclear accumulation of DAF-16::GFP in neurons and the intestine, respectively (Figures 3A, 3B, and S2D and Table S8). These results suggest that the reduced IIS pathway in neurons clearly activates DAF-16 in other tissues including the intestine, and the reduced IIS pathway in the intestine modestly activates DAF-16 in other tissues including neurons.

**Figure 4:**
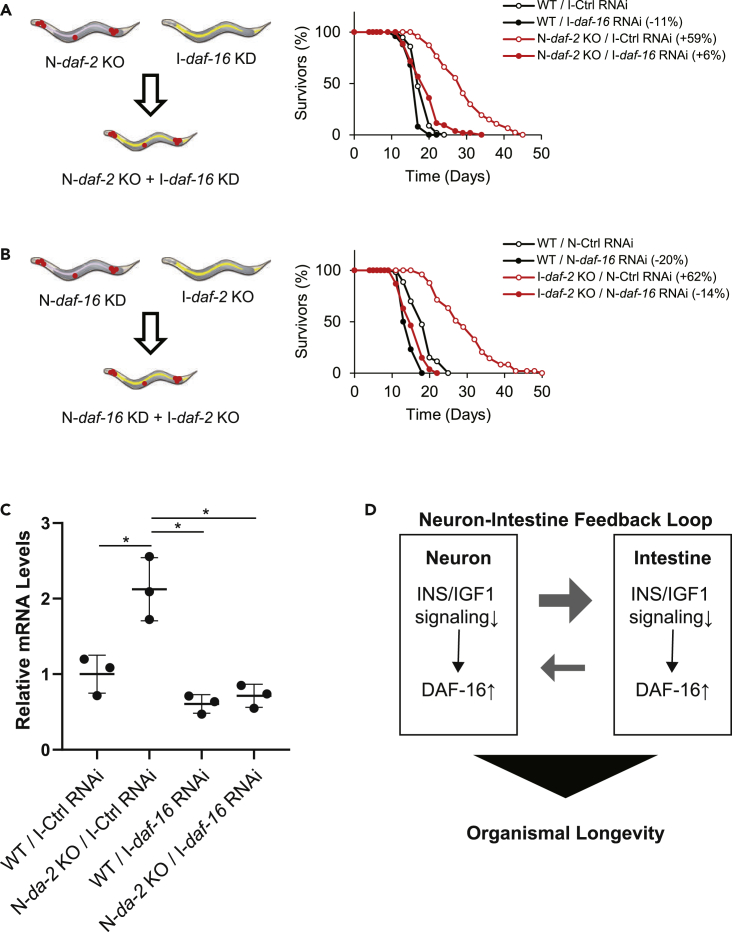
Neuron-to-intestine inter-tissue communication via INS/IGF-1 signaling pathway extends organismal lifespan (A) Scheme showing the simultaneous manipulation of two different genes (*daf-2* and *daf-16*) in two different tissues (neurons and the intestine, respectively) (left). The combination of tissue-specific knockout and knockdown enables *daf-2* to be knocked out in neurons and *daf-16* to be knocked down (see the main manuscript for details). Survival curves of the worms with deficiencies in *daf-2* in neurons and/or *daf-16* (right). Representative data from three independent experiments are shown. (B) Scheme showing the simultaneous manipulation of two different genes (*daf-16* and *daf-2*) in two different tissues (neurons and the intestine, respectively) (left). The combination of tissue-specific knockout and knockdown enables *daf-2* to be knocked out in the intestine and *daf-16* to be knocked down in neurons (see the main manuscript for details). Survival curves of the worms with deficiencies in *daf-2* in the intestine and/or *daf-16* in neurons (right). Representative data from three independent experiments are shown. (C) The qRT-PCR of *sod-3* expression in worms treated with *daf-16* RNAi in the intestine and worms whose *daf-2* was knocked out in neurons. Value represents mean ± s.d. from 3 independent experiments. Statistical significance was calculated by one-way ANOVA with a post hoc Tukey's test. ∗p < 0.05. (D) The reduction of the IIS pathway in neurons or the intestine requires the presence of DAF-16 in the intestine or neurons, respectively, to extend lifespan. Statistics are presented in Table S9.

It is possible that the inter-tissue communication between neurons and the intestine could contribute to the longevity induced by *daf-2* knockout in neurons and the intestine. To test this possibility, we needed to inhibit the activity of DAF-2 and DAF-16 in neurons and the intestine, respectively, and vice versa. Thus, we had to simultaneously manipulate two different genes in two different tissues. Firstly, we knocked out *daf-2* in neurons with a tissue-specific knockout method (Shen et al., 2014) and knocked down *daf-16* in the intestine by using an intestine-specific knockdown mutant (VP303 [Espelt et al., 2005]) (Figure 4A, left). Secondly, we knocked out *daf-2* in the intestine with a tissue-specific knockout method (Shen et al., 2014) and knocked down *daf-16* in the intestine by using a neuron-specific knockdown mutant (TU3401 [Calixto et al., 2010]) (Figure 4B, left). The obtained results demonstrated that neuronal *daf-2* knockout in the VP303 strain significantly increased lifespan, as was found in the N2 wild-type strain, and this lifespan increase was almost completely suppressed by *daf-16* knockdown in the intestine (Figure 4A, right, and Table S10) and that intestinal *daf-2* knockout in the TU3401 strain significantly increased lifespan and this lifespan increase was almost completely suppressed by *daf-16* knockdown in neurons (Figure 4B, right, and Table S9). Additionally, the increase in reproductive span induced by *daf-2* knockout in neurons was also abrogated by *daf-16* knockdown in the intestine (Figures S3A and S3B and Tables S11 and S12). Consistent with these results, the increase in *sod-3* mRNA levels by neuronal *daf-2* knockout was suppressed by intestinal *daf-16* knockdown (Figure 4C). These results indicate that inter-tissue communication between neurons and the intestine regulates the rate of organismal aging (Figure 4D). Furthermore, our results showed that growth retardation induced by *daf-2* knockout in neurons was also suppressed by *daf-16* knockdown in the intestine, suggesting that inter-tissue communication also plays a role in the regulation of the body size (Figure S3C). Overall, inter-tissue signaling between neurons and the intestine was shown to play an important role in a variety of biological processes, including aging.

### The activation of neuronal DAF-16 elicits the activation of intestinal DAF-16

**Figure 5:**
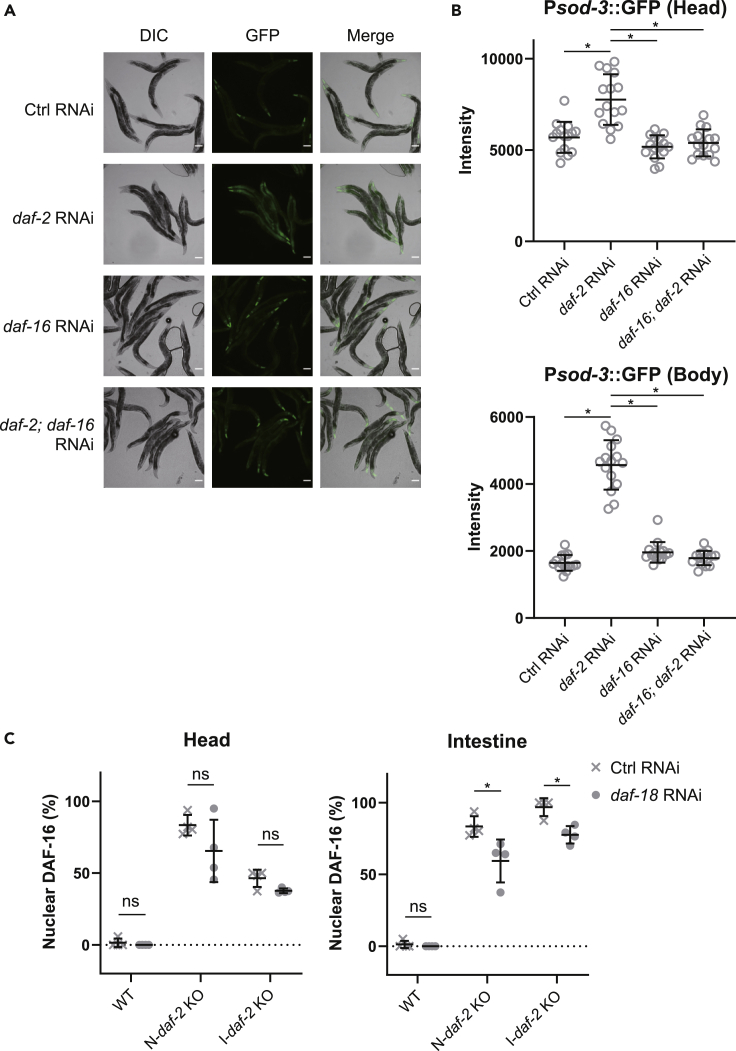
The activation of DAF-16 in neurons elicits the activation of DAF-16 in the intestine (A) Representative images showing the expression pattern of GFP driven by the promoter of *sod-3* animals treated with control, *daf-2*, *daf-16*, and *daf-2*; *daf-16* RNAi in neurons. Scale bar indicates 100 μm. (B) Quantification of GFP fluorescence intensity of head (upper) or body (lower) region of animals. Representative data from two independent experiments are shown. Data represent mean ± s.d. from 15 individual animals. Statistical significance was calculated by one-way ANOVA with a post hoc Tukey's test. ∗p < 0.05. (C) The effect of *daf-18* knockdown on the DAF-16::GFP localization in the head region (left) and the intestine (right) induced by *daf-2* knockout in neurons or the intestine. Data represent mean ± s.d. from 4 independent experiments. Statistical significance was calculated by two-way ANOVA with a post hoc Sidak's test. ∗p < 0.05. See also Figure S4. Statistics are presented in Table S13.

Our experiments show that *daf-2* deficiency in neurons induces the nuclear accumulation of DAF-16 and *sod-3* expression in the intestine (Figures 2C and S2C), implying the existence of DAF-16-to-DAF-16 inter-tissue communication from neurons to the intestine. However, the requirement of neuronal DAF-16 for communication remained unexplored. Thus, we examined whether neuronal DAF-16 is required for the induction of *sod-3* expression in the intestine, which is caused by *daf-2* deficiency in neurons. Our results showed that *daf-2* knockdown in neurons induced *sod-3* expression in the intestine and that simultaneous *daf-16* knockdown (*daf-16*; *daf-2* double knockdown) in neurons abolished *sod-3* expression in the body region as well as that in the head region (Figures 5A and 5B). These results are consistent with those of the lifespan experiments shown in Figure 1. These results thus demonstrated that DAF-16 in neurons is required for neuronal DAF-2 deficiency-induced DAF-16 activation in the intestine. They also suggest the existence of DAF-16-to-DAF-16 signaling from neurons to the intestine. Then, we investigated whether the IIS pathway in the intestine is involved in the activation of DAF-16 in the intestine in this signaling pathway. To this end, we focused on *daf-18*, a gene that encodes the *C*. *elegans* PTEN homolog (Ogg and Ruvkun, 1998), as PTEN knockdown activates the IIS pathway. The knockdown of *daf-18* suppressed the neuronal DAF-2 deficiency-induced nuclear accumulation of DAF-16::GFP in the intestine but not in the head region (Figure 5C and Table S13). Because neurons are less amenable to gene knockdown by RNAi (Kamath et al., 2003), these results suggest that the IIS pathway in tissues other than neurons, including the intestine, is involved in the increase in DAF-16::GFP nuclear accumulation induced by *daf-2* deficiency in the head region. Overall, these results suggest that *daf-2* deficiency in neurons elicits the inactivation of the IIS pathway in the intestine through neuronal DAF-16.

## Discussion

The IIS pathway is one of the most studied signaling pathways that regulate organismal lifespan (Kenyon, 2010). Tissue-specific restoration of DAF-16 to *daf-16*; *daf-2* mutants demonstrated that DAF-16 in the intestine plays a central role in lifespan regulation (Libina et al., 2003). Tissue-specific restoration of DAF-2 or AGE-1 to *daf-2* or *age-1* mutants showed that the IIS pathway in both neurons and the intestine plays a central role in lifespan regulation (Wolkow et al., 2000; Iser and Wolkow, 2007). Several studies suggested the importance of inter-tissue communication of the IIS pathway; FoxO/DAF-16-to-FoxO/DAF-16 signaling (Libina et al., 2003; Murphy et al., 2007) and FoxO/DAF-16-to-Other signaling (Zhang et al., 2013). However, the connection between neurons and the intestine remains poorly understood. Here, by manipulating two different genes in two different tissues, we have shown that the longevity induced by *daf-2* deficiency in neurons requires the presence of DAF-16 in the intestine, and vice versa. This finding demonstrates the importance of signaling between neurons and the intestine in lifespan regulation. Moreover, our results clearly show that *daf-2* deficiency in neurons or the intestine activates DAF-16 in both neurons and the intestine and that DAF-16 activation in the intestine requires the presence of DAF-16 in neurons. Taken together, our results clearly demonstrate the importance of the DAF-16-to-DAF-16 inter-tissue communication in the organismal lifespan regulation. It has previously been shown that DAF-2 and DAF-16 mainly function in different tissues to regulate lifespan, such as in neurons and the intestine, respectively, although they are two major components of the IIS pathway (Wolkow et al., 2000; Libina et al., 2003). The DAF-16-to-DAF-16 signaling feedback loop between neurons and the intestine observed in this study might explain this apparent discrepancy: our results show that the presence of DAF-16 in the intestine is required for maximizing lifespan extension induced by the reduced IIS pathway in neurons.

Our results show the existence of the DAF-16-to-DAF-16 signaling feedback loop between neurons and the intestine, in which neuronal DAF-16 may have a relatively dominant role in the loop. However, the molecular mechanisms for signal transmission between different tissues remain unsolved. The reduction of the expression of INS-7, a gene encoding an insulin-like peptide, in the intestine activates DAF-16 in neurons (Murphy et al., 2007). This signaling from the intestine to neurons could contribute to the regulation of the neuronal DAF-16 by the reduced IIS pathway in the intestine. Neither deficiency in neuronal DAF-2 nor neuropeptide release could further increase the lifespan of intestinal DAF-2 deficient animals, suggesting that both neuronal DAF-2 and neuropeptides regulate lifespan in an intestinal-DAF-16-dependent manner. However, because neither the tissue-specific KO nor *unc-31 (e928)* mutation is null, the above suggestion may not be conclusive. Our results show that deficiency in neuropeptide release synergistically increases the lifespan extension, suggesting the functional interaction between neuropeptide release and DAF-2 function; DAF-2 may regulate neuronal function to regulate distal tissues via modulating neuropeptide release, or neuropeptide release may regulate DAF-2 function in an autocrine manner.

In summary, our study reveals that IIS pathway-mediated inter-tissue communication plays a central role in lifespan regulation. Considering the importance of the IIS pathway in many biological processes, this inter-tissue communication may also participate in the systemic regulation of other processes.

### Limitations of the study

Manipulating two different genes in two different tissues by the combination of tissue-specific RNAi and tissue-specific knockout methods enabled us to examine the neuronal-DAF-16-to-intestinal-DAF-16 communication that regulates the organismal lifespan in *C*. *elegans*. We were able to verify the tissue specificity in the tissue-specific knockout experiments, but we could not rigorously demonstrate it in tissue-specific knockdown experiments because of the technical difficulties.

## STAR★Methods

### Key resources table

**Table:** 

REAGENT or RESOURCE	SOURCE	IDENTIFIER
**Bacterial and virus strains**		

*E*. *Coli*: OP50	CGC	OP50
*E*. *Coli*: HT115	CGC	HT115

**Experimental models: Organisms/strains**		

N2	CGC	N2
rrff-1(pk1417) I	CGC	NL2098
rde-1(ne219) V (outcrossed 3x); kzIs9[lin26p::nls::gfp + lin-26p::rde-1 + rol-6(su1006)]	CGC	NR222
rde-1(ne219) V (outcrossed 1x); kzIs20[hlh-1p::rde-1 + sur-5p::nls::gfp]	CGC	NR350
sid-1(pk3321) V (outcrossed 0x); uIs69[myo-2p::mCherry + unc-119p::sid-1]	CGC	TU3401
rde-1(ne219) V (outcrossed 3x); kbIs7[nhx-2p::rde-1; rol-6(su1006)]	CGC	VP303
huIs33[sod-3::gfp + rol-6(su1006)]	CGC	KN259
zIs356[daf-16::gfp + rol-6(su1006)]	CGC	TJ356
unc-31(e298) Ⅳ; dpy-11(e224) Ⅴ (outcrossed 3x)	CGC	FJ224
mkcSi13 [sun-1p::rde-1::sun-1 3′UTR + unc-119(+)] II; rde-1(ne219) V	CGC	DCL569
kyEx1700[myo-2p::mCherry + U6p::sgRNA(empty) + eft-3p::Cas9 + dpy-30p::wGxxFP(empty)]	N/A	NIS1700
kyEx1702[myo-2p::mCherry + U6p::daf-2 sgRNA + dpy-5p::Cas9 + dpy-30p::wGxxFP(daf-2)]	N/A	NIS1702
kyEx1704[myo-2p::mCherry + U6p::daf-2 sgRNA + rgef-1p::Cas9 + dpy-30p::wGxxFP(daf-2)]	N/A	NIS1704
kyEx1705[myo-2p::mCherry + U6p::daf-2 sgRNA + gly-19p::Cas9 + dpy-30p::wGxxFP(daf-2)]	N/A	NIS1705
unc-31(e298) Ⅴ (outcrossed 3x); kyEx1704[myo-2p::mCherry + U6p::daf-2 sgRNA + rgef-1p::Cas9 + dpy-30p::wGxxFP(daf-2)]	N/A	NIS1708
unc-31(e298) Ⅴ (outcrossed 3x); kyEx1705[myo-2p::mCherry + U6p::daf-2 sgRNA + gly-19p::Cas9 + dpy-30p::wGxxFP(daf-2)]	N/A	NIS1709
huIs33[sod-3::gfp + rol-6(su1006)]; kyEx1704[myo-2p::mCherry + U6p::daf-2 sgRNA + rgef-1p::Cas9]	N/A	NIS1710
huIs33[sod-3::gfp + rol-6(su1006)]; kyEx1705[myo-2p::mCherry + U6p::daf-2 sgRNA + gly-19p::Cas9]	N/A	NIS1711
huIs33[sod-3::gfp + rol-6(su1006)]; kyEx1702[myo-2p::mCherry + U6p::daf-2 sgRNA + dpy-5p::Cas9]	N/A	NIS1712
zIs356[daf-16::gfp + rol-6(su1006)]; kyEx1704[myo-2p::mCherry + U6p::daf-2 sgRNA + rgef-1p::Cas9]	N/A	NIS1713
zIs356[daf-16::gfp + rol-6(su1006)]; kyEx1705[myo-2p::mCherry + U6p::daf-2 sgRNA + gly-19p::Cas9]	N/A	NIS1714
rde-1(ne219) V (outcrossed 3x); kbIs7[nhx-2p::rde-1; rol-6(su1006)]; kyEx1705[myo-2p::mCherry + U6p::daf-2 sgRNA + rgef-1p::Cas9]	N/A	NIS1716
sid-1(pk3321) V (outcrossed 0x); uIs69[myo-2p::mCherry + unc-119p::sid-1]; kyEx1706[myo-3p::mCherry + U6p::daf-2 sgRNA + gly-19p::Cas9]	N/A	NIS1717
huIs33[sod-3::gfp + rol-6(su1006)]; kyIs1705[myo-2p::mCherry + U6p::daf-2 sgRNA + rgef-1p::Cas9] (outcrossed 0x) #1	N/A	NIS1718
huIs33[sod-3::gfp + rol-6(su1006)]; kyIs1705[myo-2p::mCherry + U6p::daf-2 sgRNA + rgef-1p::Cas9] (outcrossed 0x) #2	N/A	NIS1719
huIs33[sod-3::gfp + rol-6(su1006)]; kyIs1706[myo-2p::mCherry + U6p::daf-2 sgRNA + gly-19p::Cas9] (outcrossed 0x) #1	N/A	NIS1720
huIs33[sod-3::gfp + rol-6(su1006)]; kyIs1706[myo-2p::mCherry + U6p::daf-2 sgRNA + gly-19p::Cas9] (outcrossed 0x) #2	N/A	NIS1721
huIs33[sod-3::gfp + rol-6(su1006)]; kyIs1706[myo-2p::mCherry + U6p::daf-2 sgRNA + gly-19p::Cas9] (outcrossed 0x) #3	N/A	NIS1722
zIs356[daf-16::gfp + rol-6+C22:C40(su1006)]; kyIs1705[myo-2p::mCherry + U6p::daf-2 sgRNA + rgef-1p::Cas9] (outcrossed 0x)	N/A	NIS1723
sid-1(qt9) Ⅴ (outcrossed x1); huIs33[sod-3::gfp + rol-6(su1006)]; kyIs1705[myo-2p::mCherry + U6p::daf-2 sgRNA + rgef-1p::Cas9] (outcrossed 2x)	N/A	NIS1724
unc-31(e298) Ⅳ (outcrossed 5x)	N/A	NIS1725

**Oligonucleotides**		

For information regarding oligonucleotide sequences used in this study please refer to Table S15	This study	Table S15

**Recombinant DNA**		

Plasmid: pPD129.36 control RNAi	Addgene	#1654
Plasmid: pPD129.36 daf-2 RNAi	This study	N/A
Plasmid: pPD129.36 daf-16 RNAi	This study	N/A
Plasmid: pPD129.36 daf-16; daf-2 RNAi	This study	N/A
Plasmid: pDD162 daf-2 sgRNA, rgef-1p::Cas9	This study	N/A
Plasmid: pDD162 daf-2 sgRNA, gly-19p::Cas9	This study	N/A
Plasmid: pDD162 daf-2 sgRNA, dpy-5p::Cas9	This study	N/A
Plasmid: pDD162 gfp sgRNA, eft-3p::Cas9	This study	N/A
Plasmid: pDD162 empty sgRNA, eft-3p::Cas9	Addgene	#47549

**Software and algorithms**		

Fiji	ImgeJ	N/A
OASIS	https://sbi.postech.ac.kr/oasis/	N/A
Prism 8	GraphPad Prism	N/A
CRISPR design tool	http://crispr.mit.edu	N/A

### Resource availability

#### Lead contact

Further information and requests for resources and reagents should be directed to and will be fulfilled by the lead contact, Masaharu Uno (masaharu.uno@riken.jp).

#### Material availability

Materials used or generated in this study will be available upon reasonable request, and a material transfer agreement may be required.

#### Data and code availability

All data supporting the finding of this study are available within the paper and its supplemental information files.

### Experimental model and subject details

#### Worm cultivation

Standard *C*. *elegans* cultures were maintained using a standard protocol (Brenner, 1974). Strains were grown at 20°C on nematode growth media (NGM) plates seeded with E. coli OP50 to provide a food source. The *C*. *elegans* strains used are listed in Table S14. Before using the worms for experiments, they were bleached and passaged for two generations. The worms were synchronized by laying the eggs of approximately 10-20 fertile hermaphrodites over two hours.

### Method details

#### Lifespan measurements

Trials were conducted at 20°C without 5-fluoro-2’-deoxyuridine (FUDR) unless otherwise stated. Worms were transferred daily during the reproductive period. Mortality was scored every 2 or 3 days. Worms were scored as dead if they failed to respond to being touched by a picker. Lifespan measurements were repeated at least twice, and survival plots were generated by using single lifespan data. Single and double gene knockdown were performed with L4440 plasmid according to Kamath’s and Gouda’s methods (Kamath et al., 2003; Gouda et al., 2010).

#### Oxidative stress assay

Animals were subjected to RNAi for 4 days from the embryo stage. Adult worms were transferred individually into 60-well plate (Greiner bio-one) containing 20 μl of M9 buffer containing pro-oxidant (3 mM hydrogen peroxide) (Santoku Chemical Industries Co., Ltd). Plates were monitored almost every hour to document the number of live and dead animals. Animals were scored as dead if they failed to respond to touch with a pick.

#### Conditional knockout experiments

Conditional knockout was performed according to the protocol described by Shen et al. (Shen et al., 2014). To express Cas9 in a tissue-specific manner, we cloned several promoters (*rgef-1*, *gly-19*, *dpy-5*, and *ehn-3* promoters for neuron-, intestine-, hypodermis-, and somatic gonad-specific expression, respectively). We used the CRISPR design tool (http://crispr.mit.edu) to select the *daf-2* sgRNA.

To detect lesions induced by conditional CRISPR-Cas9, 10 transgenic worms at the L3 stage were lysed, and the DNA fragments containing the CRISPR-Cas9 targets were amplified with KOD-plus-Neo DNA polymerase (TOYOBO). The DNA fragments were purified using a PCR purification kit (Qiagen). Then, 800 ng of DNA was digested with T7 endonuclease I (NEB) and analyzed via 2% agarose gel electrophoresis.

To visualize the tissues generated with CRISPR-Cas9, we used the EGxxFP system in *C*. *elegans* (Mashiko et al., 2013). We modified pPD95.75 to generate the worm GxxFP (wGxxFP) according to the procedure described by Gouda et al. (Gouda et al., 2010). We inserted an approximately 500 bp sequence adjacent to the *daf-2* sgRNA target site in wGxxFP. The expression of the wGxxFP construct was driven by the *dpy-30* promoter (in all somatic tissues). The primers used for cloning the promoters, *daf-2* sgRNA, and wGxxFP are listed in Table S15.

#### Body size measurements

Strains were grown at 20°C for the body size measurement experiments. To synchronize the worms, 15 worms laid eggs for 2 hours. We photographed about 20 72-hour-old worms using an Olympus SZX16 camera. Then, the body size was measured using ImageJ software.

#### Dauer assay

Worms were grown at 25°C for the body dauer assay experiments. To synchronize the worms, 10 ∼ 20 worms laid eggs for 2 hours. We analyzed whether worms were dauer or not after the 72 hours post hatch.

#### Self-fertilizing reproductive span measurements

Individual synchronized L4 hermaphrodites were transferred to fresh plates every day until reproduction had ceased for at least two days. Reproductive span was defined from the first day of reproduction to the last day of viable progeny production. We defined the “Reproductive (%)” as the ratio of animals that were reproductive. The self-fertilizing reproductive span measurements were repeated at least twice. Brood size were measured every day or every two days.

#### Quantitative RT-PCR

Total RNA was isolated using TRIzol reagent (Invitrogen) in N2, VP303, TU3401, and VP303; N-*daf-2* KO worms under the indicated RNAi conditions. The extracted total RNA was reverse-transcribed into single-stranded cDNA using the ReverTra Ace qPCR RT Master Mix with gDNA Remover kit (TOYOBO) according to the manufacturer’s protocol. Quantitative RT-PCR was performed with an ABI 7300 Real-Time PCR system (Applied Biosystems) using FastStart Universal SYBR Premix Ex Taq^TM^ II (TAKARA). The relative mRNA levels were determined using the ΔΔCT method. The relative mRNA levels were normalized to that of *act-1*, a *C*. *elegans* housekeeping gene.

#### Fluorescence image analysis

The localization DAF-16::GFP and expression of P*sod-3*::*gfp* were examined using Olympus IX83 microscopy system and CellSens Dimension 1.14 software. Live worms (young adults) were mounted on 5% agar pads and immobilized with 10 mM sodium azide. For expression of P*sod-3*::*gfp* analyses, we measured the mean GFP intensity of head or body region of an individual animal using ImageJ software. For DAF-16::GFP localization analyses, we counted the number of animals whose neuronal or intestinal DAF-16 was accumulated in the nucleus. For DAPI staining, animals were fixed with 4 % PFA for 20 min and animals were mounted in VECTASHIELD (H-1200). We counted the number of cells with nuclear localization in each animal (10 animals per condition) in each experiment.

### Quantification and statistical analysis

For the lifespan assays and self-fertilizing reproductive span assays, survival graphs were generated using Excel software, and statistical tests for the significant differences between the survival curves were performed using the log-rank test with Bonferroni correction for multiple comparisons using OASIS2 software (Han et al., 2016). For the body size assays, qRT-PCR assays, or fluorescence image analyses, scatter plots were generated using GraphPad Prism 8.1 software, and statistical tests for the significant differences between the scatter plots were performed using one-way ANOVA with a post hoc Tukey’s test or two-way ANOVA with a post hoc Sidak’s test using GraphPad Prism 8.1 software (∗*P*<0.05 in every figures). We analyzed statistical significance in Figure S2A with 4 technical replicates (4 plates per condition, about 20 animals per plate) in one biological experiment using two-way ANOVA with a post hoc Sidak’s test.

## Footnotes

Supplemental information can be found online at https://doi.org/10.1016/j.isci.2021.102706.
