# Supplementary material for: Neuronal DAF-16-to-intestinal DAF-16 communication underlies organismal lifespan extension in C. elegans
Source: iScience. 2021 Jun 10;24(7):102706. doi: 10.1016/j.isci.2021.102706 (PMC8246587; doi:10.1016/j.isci.2021.102706)
Supplement: Document S1. Figures S1–S4 and Table S1–S15 [file mmc1.pdf]

**iScience, Volume *24***

## Supplemental information

# Neuronal DAF-16-to-intestinal DAF-16 communication underlies organismal lifespan extension in *C. elegans*

**Masaharu Uno, Yuri Tani, Masanori Nono, Emiko Okabe, Saya Kishimoto, Chika Takahashi, Ryoji Abe, Takuya Kurihara, and Eisuke Nishida**

**Supplemental Information**

**Neural DAF-16-to-Intestinal DAF-16 Communication Underlies Organismal Lifespan Extension in *C. elegans***

Masaharu Uno, Yuri Tani, Masanori Nono, Emiko Okabe, Saya Kishimoto, Chika Takahashi, Ryoji Abe, Takuya Kurihara, & Eisuke Nishida

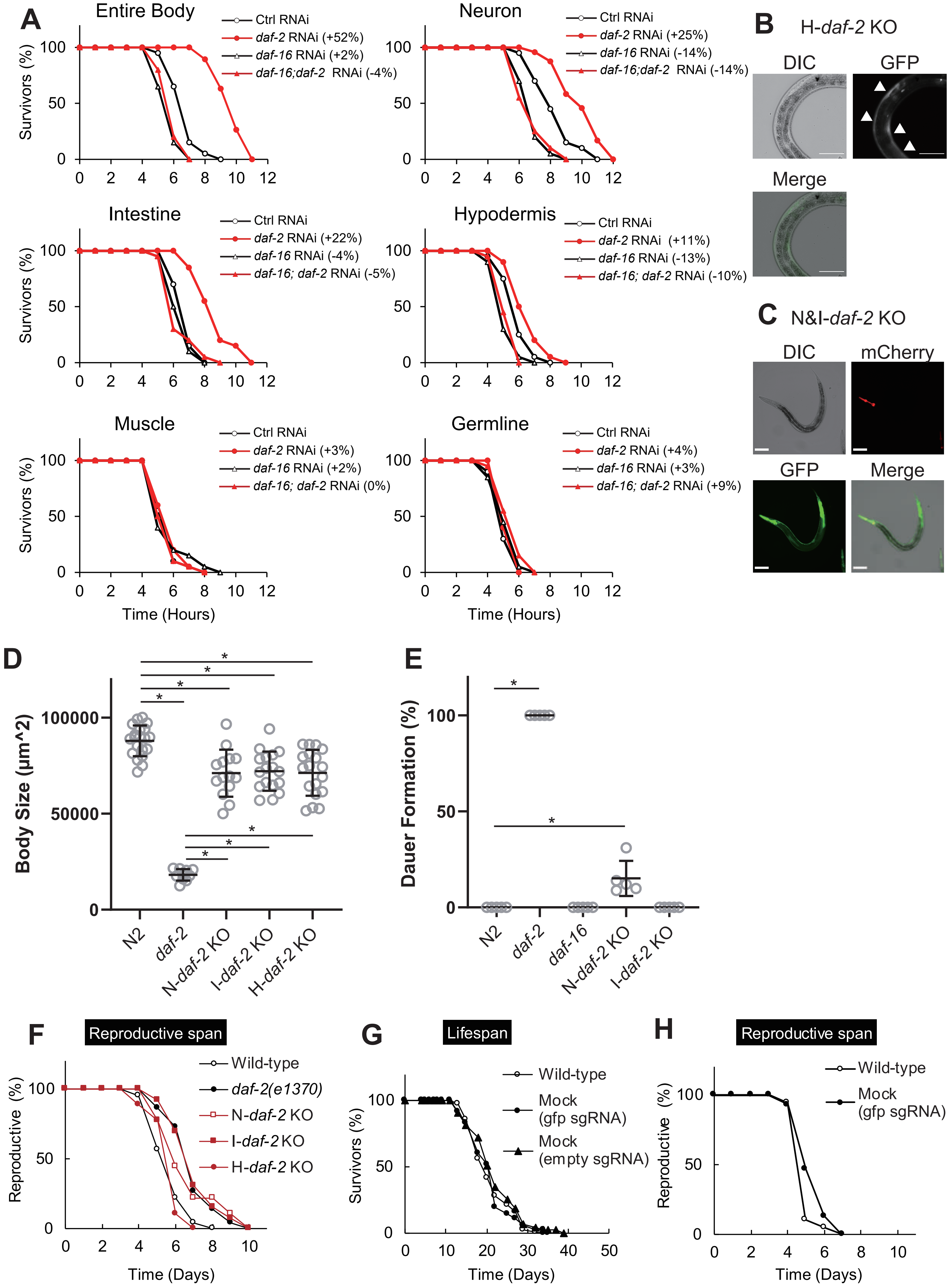

A
Entire Body
Neuron
Intestine
Hypodermis
Muscle
Germline
Ctrl RNAi
daf-2 RNAi (+52%)
daf-16 RNAi (+2%)
daf-16;daf-2 RNAi (-4%)
daf-2 RNAi (+25%)
daf-16 RNAi (-14%)
daf-16;daf-2 RNAi (-14%)
daf-2 RNAi (+22%)
daf-16 RNAi (-4%)
daf-16; daf-2 RNAi (-5%)
daf-2 RNAi (+11%)
daf-16 RNAi (-13%)
daf-16; daf-2 RNAi (-10%)
daf-2 RNAi (+3%)
daf-16 RNAi (+2%)
daf-16; daf-2 RNAi (0%)
daf-2 RNAi (+4%)
daf-16 RNAi (+3%)
daf-16; daf-2 RNAi (+9%)
Survivors (%)
Time (Hours)
B H-daf-2 KO
DIC
GFP
Merge
C N&I-daf-2 KO
DIC
mCherry
GFP
Merge
D
Body Size (μm^2)
N2
daf-2
N-daf-2 KO
I-daf-2 KO
H-daf-2 KO
E
Dauer Formation (%)
N2
daf-2
daf-16
N-daf-2 KO
I-daf-2 KO
F
Reproductive span
Wild-type
daf-2(e1370)
N-daf-2 KO
I-daf-2 KO
H-daf-2 KO
Reproductive (%)
Time (Days)
G
Lifespan
Wild-type
Mock (gfp sgRNA)
Mock (empty sgRNA)
Survivors (%)
Time (Days)
H
Reproductive span
Wild-type
Mock (gfp sgRNA)
Reproductive (%)
Time (Days)


**Figure S1**

**Figure S1 *daf-2* Knockdown in the Neuron, Intestine, and Hypodermis Increases Oxidative Stress Resistance in a Cell Autonomous DAF-16-Dependent Manner. Related to Figure 1.**

**(A)** Oxidative stress resistance in worms treated with *daf-2* and/or *daf-16* RNAi in the entire body (wild type N2, upper left), neuron (TU3401, upper middle), intestine (VP303, upper right), hypodermis (NR222, lower left), muscle (NR350, lower middle), or germline cells (NL2098, lower right). Representative data from two or three independent experiments are shown.

**(B, C)** Representative expression patterns of wGFP reconstituted from wGxxFP as observed in hypodermis *daf-2* knockout (H-*daf-2* KO, arrow heads indicate hypodermis) (B) or neuron and intestinal *daf-2* knockout (N&I-*daf-2* KO) (C) transgenic worms. Scale bar indicates 50 or 100 μm in B or C, respectively.

**(D)** Scatter plots of the body sizes of worms (N2, *daf-2(e1370)*, and neuron-, intestine-, and hypodermis-*daf-2* KO). Representative data from three independent experiments are shown. Statistical significance was calculated by one-way ANOVA with a post hoc Tukey's test. **P*<0.05. Error bars represent the mean ± s.d. Data represent size of individual animals in a representative experiment.

**(E)** Scatter plots of the dauer formation ratio from five independent experiments are shown. Error bars represent the mean ± s.d. of five independent experiments. Statistical significance was calculated by one-way ANOVA with a post hoc Dunn's test. **P*<0.05.

**(F)** Reproductive spans of worms with the knockout *daf-2* in the neuron (N-*daf-2*), intestine (I-*daf-2*), or hypodermis (H-*daf-2*). Representative data from three independent experiments are shown.

**(G)** Survival curves of worms expressing CRISPR/Cas9 and gfp sgRNA or empty sgRNA. Representative data from three independent experiments are shown.

**(H)** Reproductive spans of worms expressing CRISPR/Cas9 and gfp sgRNA. Representative

data from two independent experiments are shown.

Statistics are presented in Tables S3, 6, and 7.

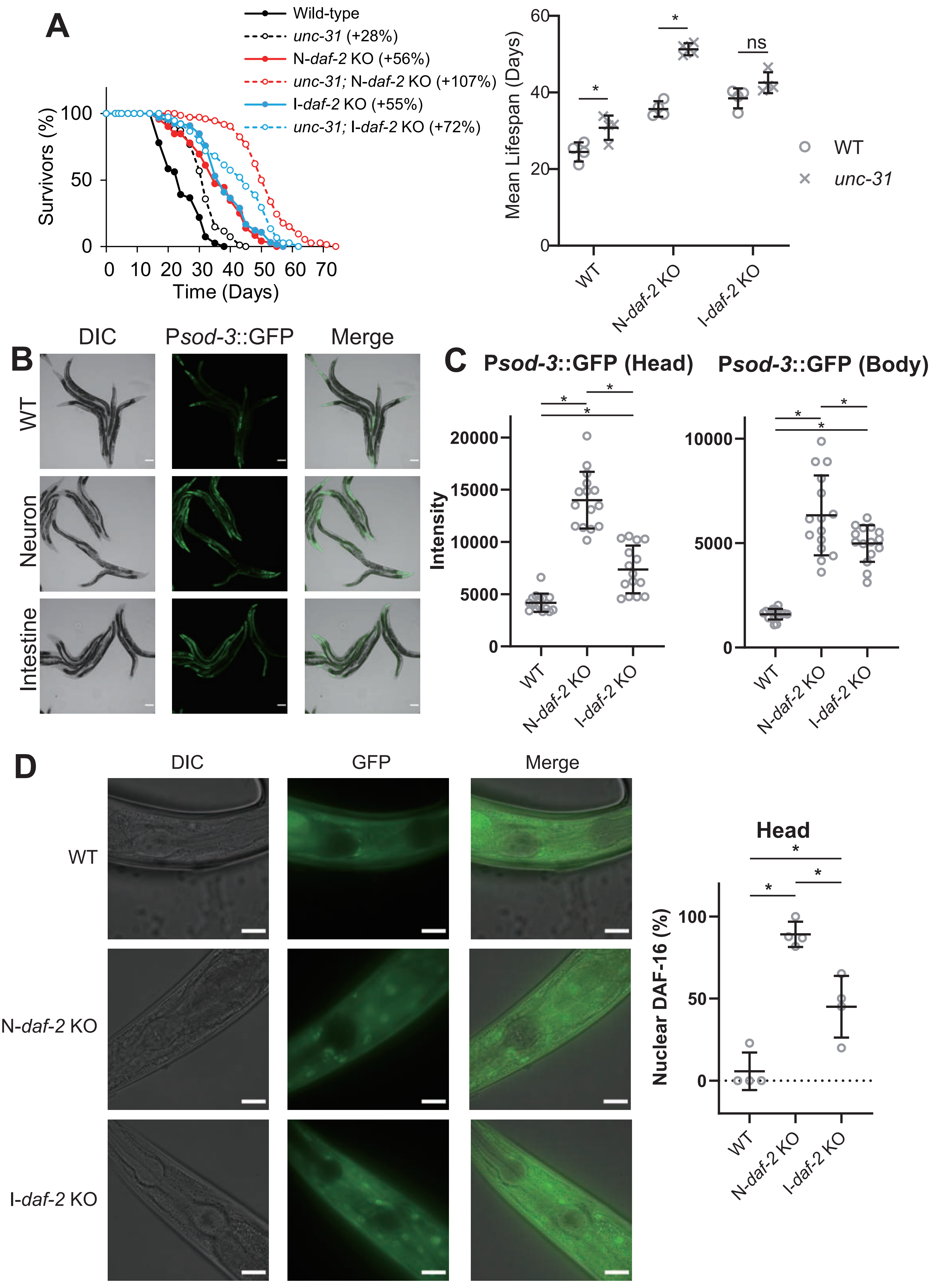
A
Wild-type
unc-31 (+28%)
N-daf-2 KO (+56%)
unc-31; N-daf-2 KO (+107%)
I-daf-2 KO (+55%)
unc-31; I-daf-2 KO (+72%)
Survivors (%)
Time (Days)
Mean Lifespan (Days)
WT
N-daf-2 KO
I-daf-2 KO
ns
unc-31
B
DIC
Psod-3::GFP
Merge
WT
Neuron
Intestine
C
Psod-3::GFP (Head)
Psod-3::GFP (Body)
Intensity
D
DIC
GFP
Merge
Head
Nuclear DAF-16 (%)

Figure S2

**Figure S2 *daf-2* Knockout in the Neuron Extends Lifespan in a UNC-31-Independent Manner. Related to Figure 2.**

**(A)** Survival curves of *unc-31(e298)* worms with the knockout of *daf-2* in the neuron or intestine (left). Mean lifespan from four technical experiments of the representative data are shown (right). To examine the functional interaction between neuron-*daf-2* KO and *unc-31* mutation, we conducted two-way ANOVA analysis with post hoc Sidak's test. (right). Statistical significance was calculated by two-way ANOVA with a post hoc Sidak's test. *$P$<0.05. Representative data from two independent experiments are shown.

**(B)** Representative images showing the expression patterns of GFP driven by the promoter of *sod-3* in wildtype (upper) and transgenic worms with knockout of the *daf-2* gene in the intestine (N-*daf-2* KO, middle) or neuron (I-*daf-2* KO, lower).

**(C)** Quantification of GFP fluorescence intensity of head (left) or body (right) region of animals. Representative data from two independent experiments are shown. Data represent mean ± s.d. from 15 individual animals. Statistical significance was calculated by one-way ANOVA with a post hoc Tukey's test. *$P$<0.05.

**(D**) Localization pattern of DAF-16::GFP in the head region of wildtype (left-upper) and that of transgenic worms with knockout of the *daf-2* gene in the neuron (N-*daf-2* KO, left-middle) or intestine (I-*daf-2* KO, left-lower). Scale bar indicates 20 μm. Ratio of the animals with DAF-16::GFP nuclear accumulation in the head neuron (right). Value represents mean ± s.d. from 4 independent experiments. Statistical significance was calculated by one-way ANOVA with a post hoc Tukey's test. *$P$<0.05.

Statistics are presented in Table S5**.**

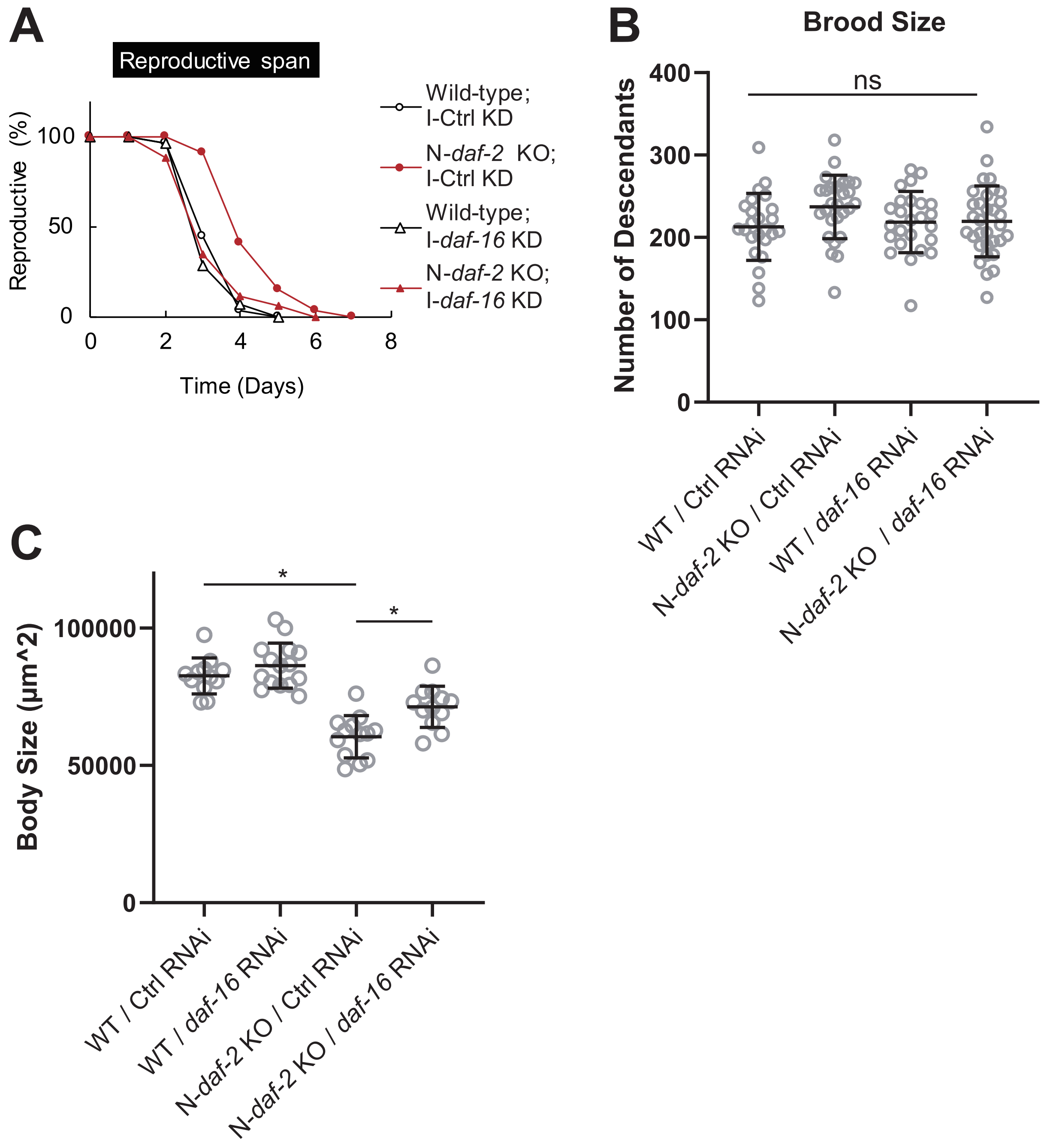
A
Reproductive span
Reproductive (%)
100
50
0
0
2
4
6
8
Time (Days)
Wild-type; I-Ctrl KD
N-daf-2 KO; I-Ctrl KD
Wild-type; I-daf-16 KD
N-daf-2 KO; I-daf-16 KD
B
Brood Size
Number of Descendants
400
300
200
100
0
ns
WT / Ctrl RNAi
N-daf-2 KO / Ctrl RNAi
WT / daf-16 RNAi
N-daf-2 KO / daf-16 RNAi
C
Body Size (μm^2)
100000
50000
0
*
*
WT / Ctrl RNAi
WT / daf-16 RNAi
N-daf-2 KO / Ctrl RNAi
N-daf-2 KO / daf-16 RNAi

**Figure S3**

**Figure S3 *daf-2* knockout in the Neuron Slows Development in an Intestinal DAF-16-Dependent Manner. Related to Figure 3.**

**(A)** Reproductive spans of worms with deficiency in *daf-2* in the neuron and /or *daf-16* in the intestine. Representative data from two independent experiments are shown. Data represent mean ± s.d. from 4 technical replicates. Statistical significance was calculated by two-way ANOVA with a post hoc Sidak's test. *$P$<0.05.

**(B)** Scatter plots of the brood size of individual worm with deficiency of *daf-2* in the neuron and/or *daf-16* in the intestine. Statistical significance was calculated by one-way ANOVA with a post hoc Tukey's test. *$P$<0.05.

**(C)** Scatter plots of the body sizes of worms with deficiency of *daf-2* in the neuron and/or *daf-16* in the intestine. Representative data from three independent experiments are shown. Statistical significance was calculated by one-way ANOVA with a post hoc Tukey's test. *$P$<0.05.

Statistics are presented in Table S11 and S12.

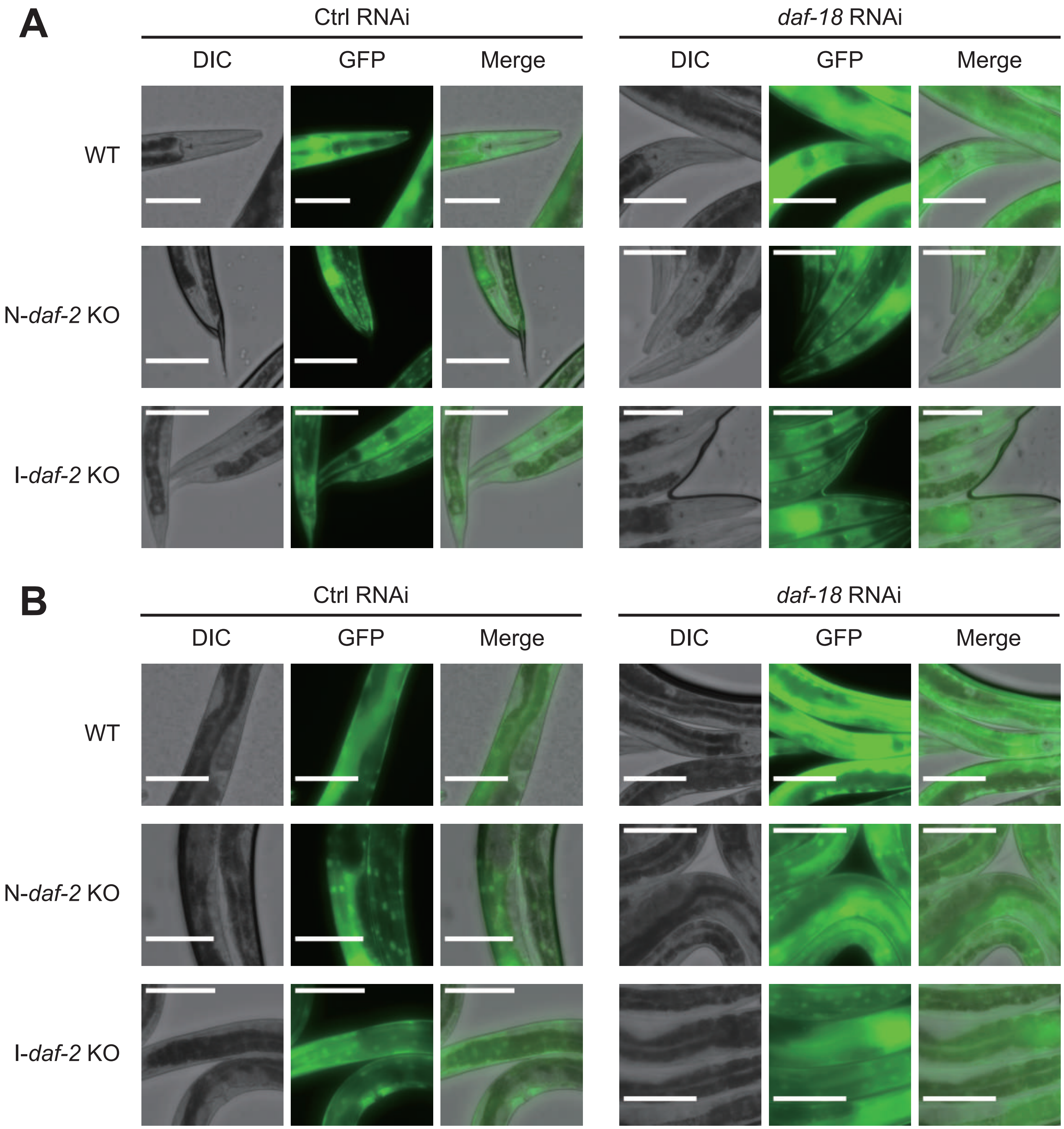
A
Ctrl RNAi
*daf-18* RNAi
DIC
GFP
Merge
DIC
GFP
Merge
WT
N-*daf-2* KO
I-*daf-2* KO
B
Ctrl RNAi
*daf-18* RNAi
DIC
GFP
Merge
DIC
GFP
Merge
WT
N-*daf-2* KO
I-*daf-2* KO

**Figure S4**

**Figure S4 DAF-16 Nuclear Accumulation in the Intestine Is Retarded by *daf-18* RNAi. Related to Figure 5.**

**(A)** Expression patterns of DAF-16::GFP in the head region of wildtype (left-upper) and that of transgenic worms with knockout of the *daf-2* gene in the neuron (N-*daf-2* KO, left-middle) or intestine (I-*daf-2* KO, left-lower). Each strain was treated with control (left) or *daf-18* (right) RNAi. Scale bar indicates 100 μm.

**(B)** Expression patterns of DAF-16::GFP in the body region of wildtype (left-upper) and that of transgenic worms with knockout of the *daf-2* gene in the intestine (N-*daf-2* KO, left-middle) or neuron (I-*daf-2* KO, left-lower). Each strain was treated with control (left) or *daf-18* (right) RNAi. Scale bar indicates 100 μm.

Statistics are presented in Table S13.

**Table S1. Statistics of the Independent Lifespan Experiments 1. Related to Figure 1**

| Trial | Strain Trial | RNAi | Average life (Days) | Std. error | Worms (n) | Bonferoni P-value | | |
|---|---|---|---|---|---|---|---|---|
| | | | | | | Ctrl vs *daf-2* | Ctrl vs *daf-16* | *daf-2* vs *daf-2;daf-16* |
| #1 | N2 | Ctrl RNAi | 19.79 | 0.59 | 26/40 | <0.0001 | 0.0053 | <0.0001 |
| | | *daf-2* RNAi | 38.5 | 0.82 | 58/72 | | | |
| | | *daf-16* RNAi | 17.36 | 0.41 | 46/80 | | | |
| | | *daf-16;daf-2* RNAi | 18.61 | 0.64 | 39/80 | | | |
| #2 | N2 | Ctrl RNAi | 19.91 | 0.35 | 75/80 | <0.0001 | <0.0001 | <0.0001 |
| | | *daf-2* RNAi | 36.47 | 0.72 | 49/60 | | | |
| | | *daf-16* RNAi | 16.47 | 0.38 | 65/80 | | | |
| | | *daf-16;daf-2* RNAi | 17.73 | 0.47 | 77/80 | | | |
| #3 | N2 | Ctrl RNAi | 26.52 | 0.48 | 69/80 | <0.0001 | 0.0001 | <0.0001 |
| | | *daf-2* RNAi | 39.41 | 0.49 | 56/80 | | | |
| | | *daf-16* RNAi | 24.02 | 0.47 | 71/80 | | | |
| | | *daf-16;daf-2* RNAi | 23.54 | 0.51 | 70/80 | | | |
| #1 | TU3401 | Ctrl RNAi | 20.19 | 0.58 | 58/60 | <0.0001 | 0.0013 | <0.0001 |
| | | *daf-2* RNAi | 32.49 | 0.6 | 55/80 | | | |
| | | *daf-16* RNAi | 17.56 | 0.53 | 48/60 | | | |
| | | *daf-16;daf-2* RNAi | 18.41 | 0.61 | 29/40 | | | |
| #2 | TU3401 | Ctrl RNAi | 21.24 | 0.41 | 59/60 | <0.0001 | <0.0001 | <0.0001 |
| | | *daf-2* RNAi | 34.08 | 0.68 | 40/40 | | | |
| | | *daf-16* RNAi | 18.46 | 0.37 | 37/40 | | | |
| | | *daf-16;daf-2* RNAi | 18.22 | 0.48 | 56/60 | | | |
| #3 | TU3401 | Ctrl RNAi | 27.86 | 0.58 | 53/60 | <0.0001 | <0.0001 | <0.0001 |
| | | *daf-2* RNAi | 36.37 | 0.44 | 54/60 | | | |
| | | *daf-16* RNAi | 23.79 | 0.58 | 55/60 | | | |
| | | *daf-16;daf-2* RNAi | 24.46 | 0.55 | 49/60 | | | |
| #1 | VP303 | Ctrl RNAi | 20.95 | 1 | 19/40 | <0.0001 | 0.0183 | <0.0001 |
| | | *daf-2* RNAi | 29.21 | 0.9 | 61/80 | | | |
| | | *daf-16* RNAi | 17.95 | 0.54 | 31/80 | | | |
| | | *daf-16;daf-2* RNAi | 20.14 | 0.59 | 32/80 | | | |
| #2 | VP303 | Ctrl RNAi | 22.57 | 0.52 | 35/40 | <0.0001 | <0.0001 | <0.0001 |
| | | *daf-2* RNAi | 32.22 | 1.09 | 18/40 | | | |
| | | *daf-16* RNAi | 19.59 | 0.34 | 54/60 | | | |
| | | *daf-16;daf-2* RNAi | 20.33 | 0.54 | 55/60 | | | |
| #3 | VP303 | Ctrl RNAi | 26.53 | 0.57 | 47/60 | <0.0001 | 0.0025 | <0.0001 |
| | | *daf-2* RNAi | 31.66 | 0.65 | 41/60 | | | |
| | | *daf-16* RNAi | 24.55 | 0.52 | 31/40 | | | |
| | | *daf-16;daf-2* RNAi | 24.46 | 0.48 | 45/60 | | | |
| #1 | NR222 | Ctrl RNAi | 19.44 | 0.42 | 68/80 | <0.0001 | 0.0566 | <0.0001 |
| | | *daf-2* RNAi | 23.17 | 0.74 | 37/40 | | | |
| | | *daf-16* RNAi | 17.85 | 0.45 | 58/80 | | | |
| | | *daf-16;daf-2* RNAi | 18.46 | 0.45 | 68/80 | | | |
| #2 | NR222 | Ctrl RNAi | 19.89 | 0.57 | 39/60 | <0.0001 | 0.0122 | <0.0001 |
| | | *daf-2* RNAi | 24.74 | 0.57 | 57/60 | | | |
| | | *daf-16* RNAi | 17.33 | 0.55 | 51/60 | | | |
| | | *daf-16;daf-2* RNAi | 17.79 | 0.67 | 53/60 | | | |
| #3 | NR222 | Ctrl RNAi | 25.9 | 0.84 | 38/60 | 0.0012 | 0.0065 | <0.0001 |
| | | *daf-2* RNAi | 29.8 | 0.66 | 50/60 | | | |
| | | *daf-16* RNAi | 22.9 | 0.73 | 45/60 | | | |
| | | *daf-16;daf-2* RNAi | 23.15 | 0.83 | 47/60 | | | |
| #1 | DCL569 | Ctrl RNAi | 15.62 | 0.2 | 76/80 | <0.0001 | 1 | <0.0001 |
| | | *daf-2* RNAi | 18.5 | 0.36 | 72/81 | | | |
| | | *daf-16* RNAi | 15.31 | 0.24 | 73/80 | | | |
| | | *daf-16;daf-2* RNAi | 15.63 | 0.22 | 78/80 | | | |
| #2 | DCL569 | Ctrl RNAi | 15.96 | 0.2 | 63/78 | <0.0001 | <0.0001 | <0.0001 |
| | | *daf-2* RNAi | 18.68 | 0.38 | 72/80 | | | |
| | | *daf-16* RNAi | 14.32 | 0.22 | 69/80 | | | |
| | | *daf-16;daf-2* RNAi | 15.53 | 0.25 | 78/80 | | | |
| #1 | NR350 | Ctrl RNAi | 17.56 | 0.55 | 27/40 | 1 | 1 | 1 |
| | | *daf-2* RNAi | 17.41 | 0.57 | 25/40 | | | |
| | | *daf-16* RNAi | 17.23 | 0.35 | 45/80 | | | |
| | | *daf-16;daf-2* RNAi | 17 | 0.48 | 32/40 | | | |
| #2 | NR350 | Ctrl RNAi | 17.62 | 0.35 | 52/60 | 0.2851 | 1 | 0.0039 |
| | | *daf-2* RNAi | 18.85 | 0.56 | 26/31 | | | |
| | | *daf-16* RNAi | 17.29 | 0.83 | 14/20 | | | |
| | | *daf-16;daf-2* RNAi | 16.58 | 0.35 | 40/60 | | | |

**Table S2. Statistics of the Independent Oxidative Sress Experiments 1. Related to Figure S1**

| Trial | Strain Trial | RNAi | Average life (Hours) | Std. error | Worms (n) | Bonferoni P-value | | |
|---|---|---|---|---|---|---|---|---|
| | | | | | | Ctrl vs *daf-2* | Ctrl vs *daf-16* | *daf-2* vs *daf-2;daf-16* |
| 1 | **N2** | **Ctrl RNAi** | 4.25 | 0.10 | 20/20 | 1.6e-9 | 1 | 0 |
| | | ***daf-2* RNAi** | 6.45 | 0.22 | 20/20 | | | |
| | | ***daf-16* RNAi** | 4.35 | 0.11 | 20/20 | | | |
| | | ***daf-16;daf-2* RNAi** | 4.1 | 0.07 | 20/20 | | | |
| 2 | **N2** | **Ctrl RNAi** | 6.5 | 0.21 | 20/20 | 5.5e-6 | 1 | 9.5e-8 |
| | | ***daf-2* RNAi** | 8.55 | 0.25 | 20/20 | | | |
| | | ***daf-16* RNAi** | 6.3 | 0.26 | 20/20 | | | |
| | | ***daf-16;daf-2* RNAi** | 6 | 0.19 | 20/20 | | | |
| 3 | **N2** | **Ctrl RNAi** | 6.8 | 0.19 | 20/20 | 7.8e-9 | 0.0013 | 0 |
| | | ***daf-2* RNAi** | 9.79 | 0.22 | 20/20 | | | |
| | | ***daf-16* RNAi** | 5.8 | 0.15 | 20/20 | | | |
| | | ***daf-16;daf-2* RNAi** | 6 | 0.14 | 20/20 | | | |
| 1 | **TU3401** | **Ctrl RNAi** | 7 | 0.41 | 20/20 | 0.019 | 1 | 0.0002 |
| | | ***daf-2* RNAi** | 8.85 | 0.28 | 20/20 | | | |
| | | ***daf-16* RNAi** | 6.85 | 0.22 | 20/20 | | | |
| | | ***daf-16;daf-2* RNAi** | 7.35 | 0.19 | 20/20 | | | |
| 2 | **TU3401** | **Ctrl RNAi** | 5.25 | 0.10 | 20/20 | 0 | 0.07 | 4.9e-8 |
| | | ***daf-2* RNAi** | 6.95 | 0.05 | 20/20 | | | |
| | | ***daf-16* RNAi** | 5.7 | 0.16 | 20/20 | | | |
| | | ***daf-16;daf-2* RNAi** | 5.4 | 0.15 | 20/20 | | | |
| 3 | **TU3401** | **Ctrl RNAi** | 8.06 | 0.24 | 20/20 | 1.4e-6 | 0.0042 | 0 |
| | | ***daf-2* RNAi** | 10.08 | 0.22 | 20/20 | | | |
| | | ***daf-16* RNAi** | 6.95 | 0.18 | 20/20 | | | |
| | | ***daf-16;daf-2* RNAi** | 6.9 | 0.22 | 20/20 | | | |
| 1 | **VP303** | **Ctrl RNAi** | 6.2 | 0.11 | 20/20 | 2.3e-5 | 0.004 | 0.0041 |
| | | ***daf-2* RNAi** | 7.6 | 0.23 | 20/20 | | | |
| | | ***daf-16* RNAi** | 7 | 0.18 | 20/20 | | | |
| | | ***daf-16;daf-2* RNAi** | 6.25 | 0.25 | 20/20 | | | |
| 2 | **VP303** | **Ctrl RNAi** | 7.35 | 0.19 | 20/20 | 3.5e-8 | 0.0005 | 0 |
| | | ***daf-2* RNAi** | 9.55 | 0.11 | 20/20 | | | |
| | | ***daf-16* RNAi** | 8.55 | 0.13 | 20/20 | | | |
| | | ***daf-16;daf-2* RNAi** | 7.25 | 0.12 | 20/20 | | | |
| 3 | **VP303** | **Ctrl RNAi** | 6.85 | 0.15 | 20/20 | 3.5e-8 | 0.0005 | 0 |
| | | ***daf-2* RNAi** | 8.35 | 0.20 | 17/17 | | | |
| | | ***daf-16* RNAi** | 6.55 | 0.17 | 20/20 | | | |
| | | ***daf-16;daf-2* RNAi** | 6.5 | 0.22 | 20/20 | | | |
| 1 | **NR222** | **Ctrl RNAi** | 6 | 0.20 | 20/20 | 0.14 | 0.017 | 0.0001 |
| | | ***daf-2* RNAi** | 6.65 | 0.23 | 20/20 | | | |
| | | ***daf-16* RNAi** | 5.25 | 0.16 | 20/20 | | | |
| | | ***daf-16;daf-2* RNAi** | 5.4 | 0.13 | 20/20 | | | |
| 2 | **NR222** | **Ctrl RNAi** | 6.3 | 0.14 | 20/20 | 0.017 | 0.17 | 3.5e-5 |
| | | ***daf-2* RNAi** | 7 | 0.16 | 20/20 | | | |
| | | ***daf-16* RNAi** | 6.7 | 0.16 | 20/20 | | | |
| | | ***daf-16;daf-2* RNAi** | 6.1 | 0.07 | 20/20 | | | |
| 3 | **NR222** | **Ctrl RNAi** | 6.9 | 0.19 | 20/20 | 0.24 | 1.80E-05 | 2.2e-6 |
| | | ***daf-2* RNAi** | 7.45 | 0.26 | 20/20 | | | |
| | | ***daf-16* RNAi** | 5.45 | 0.17 | 20/20 | | | |
| | | ***daf-16;daf-2* RNAi** | 5.6 | 0.13 | 20/20 | | | |
| 1 | **NL2098** | **Ctrl RNAi** | 6.5 | 0.15 | 20/20 | 1 | 0.16 | 0.88 |
| | | ***daf-2* RNAi** | 6.35 | 0.11 | 20/20 | | | |
| | | ***daf-16* RNAi** | 7 | 0.19 | 20/20 | | | |
| | | ***daf-16;daf-2* RNAi** | 6.2 | 0.09 | 20/20 | | | |
| 2 | **NL2098** | **Ctrl RNAi** | 5.2 | 0.13 | 20/20 | 0.98 | 1 | 0.52 |
| | | ***daf-2* RNAi** | 5.4 | 0.11 | 20/20 | | | |
| | | ***daf-16* RNAi** | 5.35 | 0.18 | 20/20 | | | |
| | | ***daf-16;daf-2* RNAi** | 5.65 | 0.18 | 20/20 | | | |
| 1 | **NR350** | **Ctrl RNAi** | 4.35 | 0.16 | 20/20 | 1 | 1 | 0.39 |
| | | ***daf-2* RNAi** | 4.2 | 0.09 | 20/20 | | | |
| | | ***daf-16* RNAi** | 4.3 | 0.12 | 20/20 | | | |
| | | ***daf-16;daf-2* RNAi** | 4.45 | 0.13 | 20/20 | | | |
| 2 | **NR350** | **Ctrl RNAi** | 5.7 | 0.17 | 20/20 | 1 | 1 | 1 |
| | | ***daf-2* RNAi** | 5.85 | 0.19 | 20/20 | | | |
| | | ***daf-16* RNAi** | 5.8 | 0.27 | 20/20 | | | |
| | | ***daf-16;daf-2 RNAi*** | 5.65 | 0.18 | 20/20 | | | |

**Table S3. (A) Statistics of the Independent Dauer Assay. Related to Figure S1**

| | N2 | | *daf-2* | | *daf-16* | | N-*daf-2* KO | | I-*daf-2* KO | | N2 | *daf-2* | *daf-16* | N-*daf-2* KO | I-*daf-2* KO |
|---|---|---|---|---|---|---|---|---|---|---|---|---|---|---|---|
| | normal | dauer | normal | dauer | normal | dauer | normal | dauer | normal | dauer | | | | | |
| Exp #1 | 196 | 0 | 0 | 78 | 158 | 0 | 60 | 27 | 88 | 0 | 0 | 100 | 0 | 31.0 | 0 |
| Exp #2 | 70 | 0 | 0 | 31 | 73 | 0 | 54 | 8 | 35 | 0 | 0 | 100 | 0 | 12.9 | 0 |
| | 62 | 0 | 0 | 29 | 63 | 0 | 47 | 4 | 46 | 0 | 0 | 100 | 0 | 7.8 | 0 |
| | 67 | 0 | 0 | 28 | 66 | 0 | 45 | 8 | 40 | 0 | 0 | 100 | 0 | 15.1 | 0 |
| Exp #3 | 108 | 0 | 0 | 25 | 83 | 0 | 91 | 12 | 105 | 0 | 0 | 100 | 0 | 11.7 | 0 |
| | 114 | 0 | 0 | 28 | 90 | 0 | 105 | 7 | 105 | 0 | 0 | 100 | 0 | 6.3 | 0 |
| Exp #4 | 132 | 0 | 0 | 32 | 130 | 0 | 80 | 15 | 222 | 0 | 0 | 100 | 0 | 15.8 | 0 |
| | 129 | 0 | 0 | 50 | 133 | 0 | 75 | 10 | 163 | 0 | 0 | 100 | 0 | 11.8 | 0 |
| Exp #5 | 83 | 0 | 0 | 30 | 121 | 0 | 97 | 11 | 142 | 0 | 0 | 100 | 0 | 10.2 | 0 |
| | 82 | 0 | 0 | 49 | 103 | 0 | 60 | 6 | 133 | 0 | 0 | 100 | 0 | 9.1 | 0 |
| | n = 1043 | | n = 380 | | n = 1020 | | n = 822 | | n = 1079 | | 0 ± 0 | 100 ± 0 | 0 ± 0 | 13 ± 7.0 | 0 ± 0 |

**Table S3. (B) Statistics of the Independent Dauer Assay. Related to Figure S1**

| | |
|---|---|
| ANOVA summary | |
| F | 571.3 |
| P value | <0.0001 |
| P value summary | **** |
| Significant diff. among means (P < 0.( | Yes |
| R square | 0.9913 |

| **Multiple comparison (Dunn's multiple comparisons test)** | | | | |
|---|---|---|---|---|
| Dunn's multiple comparisons test | Mean rank diff. | Significant? | Summary | Adjusted P Value |
| N2 vs. *daf-2* | -30 | Yes | **** | <0.0001 |
| N2 vs. *daf-16* | 0 | No | ns | >0.9999 |
| N2 vs. N-*daf-2* KO | -20 | Yes | ** | 0.005 |
| N2 vs. I-*daf-2* KO | 0 | No | ns | >0.9999 |
| *daf-2* vs. *daf-16* | 30 | Yes | **** | <0.0001 |
| *daf-2* vs. N-*daf-2* KO | 10 | No | ns | 0.8168 |
| *daf-2* vs. I-*daf-2* KO | 30 | Yes | **** | <0.0001 |
| *daf-16* vs. N-*daf-2* KO | -20 | Yes | ** | 0.005 |
| *daf-16* vs. I-*daf-2* KO | 0 | No | ns | >0.9999 |
| N-*daf-2* KO vs. I-*daf-2* KO | 20 | Yes | ** | 0.005 |

**Table S4. Statistics of the Independent Lifespan Experiments 2. Related to Figure 1**

| Trial | Strain | Mean lifespan (Days) | Std. error | Worms (n) | Bonferroni P-value | | | | |
|---|---|---|---|---|---|---|---|---|---|
| | | | | | vs WT | vs *daf-2(e1370)* | vs neuronal *daf-2* KO | vs intestinal *daf-2* KO | vs hypodermal *daf-2* KO |
| # 1 | **WT** | 21.51 | 0.46 | 61 / 101 | - | - | < 0.0001 | < 0.0001 | 0.0218 |
| | **Neuronal *daf-2* KO** | 36.47 | 2.32 | 26 / 80 | | | - | 0.0848 | < 0.0001 |
| | **Intestinal *daf-2* KO** | 32.32 | 1.16 | 55 / 80 | | | | - | < 0.0001 |
| | **Hypodermal *daf-2* KO** | 23.32 | 1.07 | 34 / 80 | | | | | - |
| # 2 | **WT** | 22.05 | 0.36 | 75 / 100 | - | < 0.0001 | < 0.0001 | < 0.0001 | 0.2221 |
| | ***daf-2(e1370)*** | 43.27 | 2.15 | 54 / 100 | | - | 0.0001 | 0.0001 | < 0.0001 |
| | **Neuronal *daf-2 KO*** | 33.58 | 1.59 | 47 / 80 | | | - | 0.5565 | < 0.0001 |
| | **Intestinal *daf-2* KO** | 34.83 | 1.44 | 61 / 81 | | | | - | < 0.0001 |
| | **Hypodermal *daf-2* KO** | 22.63 | 0.67 | 48 / 80 | | | | | - |
| # 3 | **WT** | 20.94 | 0.54 | 62 / 100 | - | < 0.0001 | < 0.0001 | < 0.0001 | < 0.0001 |
| | ***daf-2(e1370)*** | 42.92 | 2.48 | 39 / 80 | | - | 1 | 0.9405 | 0.003 |
| | **Neuronal *daf-2 KO*** | 47.26 | 2.27 | 45 / 80 | | | - | 0.0278 | < 0.0001 |
| | **Intestinal *daf-2* KO** | 40.24 | 1.44 | 52 / 80 | | | | - | 0.0029 |
| | **Hypodermal *daf-2* KO** | 31.64 | 1.91 | 21 / 80 | | | | | - |

**Table S5. (A) Statistics of the Independent Lifespan Experiments 3_1 (log-rank test with Bonferroni correction). Related to Figure 1 and S2**

| Trial | Strain | Mean lifespan (Days) | Std. error | Worms (n) | Bonferroni P-value | | | | | | | |
|---|---|---|---|---|---|---|---|---|---|---|---|---|
| | | | | | vs WT | vs *daf-2(e1370)* | vs Neuronal *daf-2* KO | vs Intestinal *daf-2* KO | vs Neuronal & Intestinal *daf-2* KO | vs *unc-31(e298)* | vs *unc-31(e298);* neuronal *daf-2* KO | vs *unc-31(e298);* intestinal *daf-2* KO |
| # 1 | WT | 24.73 | 0.97 | 41 / 100 | - | < 0.0001 | < 0.0001 | < 0.0001 | < 0.0001 | < 0.0001 | < 0.0001 | < 0.0001 |
| | *daf-2(e1370)* | 49.79 | 1.5 | 67 / 78 | | - | < 0.0001 | < 0.0001 | < 0.0001 | < 0.0001 | 1 | 0.0002 |
| | Neuronal *daf-2* KO | 38.68 | 1.15 | 72 / 80 | | | - | 1 | 0.0176 | < 0.0001 | < 0.0001 | 0.0187 |
| | Intestinal *daf-2* KO | 38.38 | 1.09 | 66 / 80 | | | | - | 0.0384 | < 0.0001 | < 0.0001 | 0.0106 |
| | Neuronal & intestinal *daf-2* KO | 33.25 | 1.17 | 62 / 78 | | | | | - | 0.3257 | < 0.0001 | < 0.0001 |
| | *unc-31(e298)* | 31.63 | 0.61 | 95 / 100 | | | | | | - | < 0.0001 | < 0.0001 |
| | *unc-31(e298);* neuronal *daf-2* KO | 51.23 | 1.03 | 74 / 80 | | | | | | | - | 0.0001 |
| | *unc-31(e298);* intestinal *daf-2* KO | 42.53 | 1.29 | 75 / 81 | | | | | | | | - |
| # 2 | WT | 22.97 | 0.87 | 37 /102 | - | < 0.0001 | < 0.0001 | < 0.0001 | 0.0072 | < 0.0001 | 0.2221 | 0.1035 |
| | *daf-2(e1370)* | 55.27 | 1.46 | 48 / 62 | | - | < 0.0001 | < 0.0001 | < 0.0001 | < 0.0001 | 0.2564 | 0.0001 |
| | Neuronal *daf-2 KO* | 37.71 | 1.08 | 68 / 81 | | | - | 0.0001 | < 0.0001 | 0.0001 | < 0.0001 | < 0.0001 |
| | Intestinal *daf-2* KO | 40.96 | 1.02 | 57 / 80 | | | | - | < 0.0001 | 0.5565 | < 0.0001 | < 0.0001 |
| | Neuronal & intestinal *daf-2* KO | 27.62 | 0.96 | 66 / 78 | | | | | - | 0.2035 | < 0.0001 | < 0.0001 |
| | *unc-31(e298)* | 30.82 | 0.67 | 98 / 102 | | | | | | - | < 0.0001 | < 0.0001 |
| | *unc-31(e298);* neuronal *daf-2* KO | 51.85 | 1.07 | 75 / 80 | | | | | | | - | 0.4515 |
| | *unc-31(e298);* intestinal *daf-2* KO | 45.27 | 1.29 | 71 / 81 | | | | | | | | - |
| # 3 | WT | 27.8 | 0.87 | 50 / 101 | - | < 0.0001 | < 0.0001 | < 0.0001 | < 0.0001 | | | |
| | *daf-2(e1370)* | 48.9 | 2.17 | 33 / 78 | | - | 0.0747 | 0.0712 | 1 | | | |
| | Neuronal *daf-2* KO | 42.82 | 1.28 | 53 / 80 | | | - | 1 | 0.0027 | | | |
| | Intestinal *daf-2* KO | 41.1 | 1.43 | 10 / 79 | | | | - | 0.0011 | | | |
| | Neuronal & Intestinal *daf-2 KO* | 49.44 | 1.34 | 40 / 74 | | | | | - | | | |

**Table S5. (B) Statistics of the Independent Lifespan Experiments 3_2 (2-way ANOVA post hoc Sidak's test). Related to Figure 1 and S2**

| Trial | Sidak's multiple comparisons test | Mean Diff. | 95.00% CI of diff. | Significant? | Summary | Adjusted P Value |
|---|---|---|---|---|---|---|
| #1 | **WT vs *unc-31*** | -6.325 | -10.94 to -1.713 | Yes | ** | 0.006 |
| | **N-*daf-2* KO vs *unc-31;* N-*daf-2* KO** | -15.59 | -20.20 to -10.98 | Yes | **** | <0.0001 |
| | **I-*daf-2* KO vs *unc-31;* I-*daf-2* KO** | -4.1 | -8.712 to 0.5120 | No | ns | 0.0903 |
| #2 | **WT vs *unc-31*** | -7.818 | -11.60 to -4.036 | Yes | *** | 0.0001 |
| | **N-*daf-2* KO vs *unc-31;* N-*daf-2* KO** | -14.21 | -17.99 to -10.42 | Yes | **** | <0.0001 |
| | **I-*daf-2* KO vs *unc-31;* I-*daf-2* KO** | -3.218 | -6.999 to 0.5644 | No | ns | 0.1099 |

| | #1 | **WT** | | | | ***unc-31*** | | | |
|---|---|---|---|---|---|---|---|---|---|
| Mean lifespan of replicates | **WT** | 25.41 (n=25) | 24.35 (n=25) | 21.13 (n=25) | 27 (n=25) | 33.81 (n=25) | 31.45 (n=25) | 31.6 (n=25) | 26.33 (n=25) |
| | **N-*daf-2* KO** | 36.1 (n=20) | 34.32 (n=20) | 34 (n=20) | 38.37 (n=20) | 49.68 (n=20) | 53.06 (n=20) | 52.12 (n=20) | 50.3 (n=20) |
| | **I-*daf-2* KO** | 40.33 (n=20) | 38.97 (n=20) | 39.88 (n=20) | 34.65 (n=20) | 41.8 (n=20) | 46.56 (n=20) | 40.47 (n=21) | 41.4 (n=20) |

| | #2 | **WT** | | | | ***unc-31*** | | | |
|---|---|---|---|---|---|---|---|---|---|
| Mean lifespan of replicates | **WT** | 24.91 (n=26) | 24.33 (n=26) | 23.27 (n=25) | 19 (n=25) | 29.05 (n=25) | 31.95 (n=26) | 30.89 (n=26) | 30.89 (n=25) |
| | **N-*daf-2* KO** | 39.39 (n=21) | 35.2 (n=20) | 37.67 (n=20) | 38.44 (n=20) | 50.16 (n=20) | 53.94 (n=20) | 50.68 (n=20) | 52.74 (n=20) |
| | **I-*daf-2* KO** | 40.89 (n=20) | 42.77 (n=21) | 40.83 (n=20) | 39.38 (n=20) | 48.28 (n=21) | 42.1 (n=20) | 43.74 (n=20) | 42.62 (n=20) |

**Table S6. Statistics of the Independent Reproductive Span Experiments 1. Related to Figure S1**

| Trial | Strain | Mean reproductive lifespan (Days) | Std. error | Worms | Bonferroni P-value | | | | | | |
|---|---|---|---|---|---|---|---|---|---|---|---|
| | | | | | vs WT | vs *daf-2(e1370)* | vs Neuronal *daf-2* KO | vs Intestinal *daf-2* KO | vs Hypodermal *daf-2* KO | vs Somatic gonadal *daf-2* KO | vs mock (gfp sgRNA) |
| # 1 | **WT** | 5.11 | 0.13 | 19 / 20 | - | 0.0001 | 0.0001 | 0.0052 | 1 | | |
| | ***daf-2(e1370)*** | 6.53 | 0.25 | 15 / 20 | | - | 0.1538 | 1 | 0.0201 | | |
| | **Neuronal *daf-2* KO** | 7.86 | 0.47 | 7 / 20 | | | - | 1 | 0.0047 | | |
| | **Intestinal *daf-2* KO** | 7 | 0.53 | 6 / 20 | | | | - | 0.0756 | | |
| | **Hypodermal *daf-2* KO** | 5.25 | 0.27 | 12 / 20 | | | | | - | | |
| # 2 | **WT** | 5.32 | 0.09 | 24 / 30 | - | <0.0001 | <0.0001 | <0.0001 | 0.0596 | 0.1728 | 0.075 |
| | ***daf-2(e1370)*** | 7.44 | 0.33 | 21 / 30 | | - | 0.9872 | 1 | 0.0604 | 0.0274 | 1 |
| | **Neuronal *daf-2* KO** | 8.29 | 0.43 | 9 / 30 | | | - | 1 | 0.0047 | 0.0018 | 0.0912 |
| | **Intestinal *daf-2* KO** | 8.75 | 0.88 | 10 / 30 | | | | - | 0.0168 | 0.0032 | 0.1272 |
| | **Hypodermal *daf-2* KO** | 6.17 | 0.29 | 11 / 30 | | | | | - | 1 | 1 |
| | **Somatic gonadal *daf-2* KO** | 6.16 | 0.29 | 22 / 30 | | | | | | - | 1 |
| | **mock (gfp sgRNA)** | 6.67 | 0.45 | 17 / 30 | | | | | | | - |
| # 3 | **WT** | 5.81 | 0.19 | 23/ 30 | - | 0.0001 | 0.0001 | 0.0003 | 0.4365 | 0.6741 | 1 |
| | ***daf-2(e1370)*** | 7.41 | 0.28 | 22 / 30 | | - | 1 | 1 | 0.2731 | 0.4672 | 0.0001 |
| | **Neuronal *daf-2* KO** | 8.19 | 0.47 | 17 / 30 | | | - | 1 | 0.3932 | 0.0683 | 0.0003 |
| | **Intestinal *daf-2* KO** | 7.72 | 0.38 | 9 / 30 | | | | - | 0.3013 | 0.2511 | 0.0002 |
| | **Hypodermal *daf-2* KO** | 6.23 | 0.2 | 13 / 30 | | | | | - | 1 | 0.369 |
| | **Somatic gonadal *daf-2* KO** | 6.33 | 0.31 | 9 / 30 | | | | | | - | 0.7356 |
| | **mock (gfp sgRNA)** | 5.83 | 0.16 | 22/29 | | | | | | | - |

**Table S7. Statistics of the Independent Lifespan Experiments 4. Related to Figure S1**

| Trial | Strain | Mean lifespan (Days) | Std. error | Worms (n) | Bonferroni P-value | | |
|---|---|---|---|---|---|---|---|
| | | | | | vs WT | vs mock (empty sgRNA) | vs mock (gfp sgRNA) |
| # 1 | **WT** | 21.39 | 0.57 | 76 / 100 | - | 0.797 | 1 |
| | **mock (empty sgRNA)** | 22.11 | 0.9 | 43 / 100 | | - | 0.808 |
| | **mock (gfp sgRNA)** | 20.99 | 0.85 | 35 / 100 | | | - |
| # 2 | **WT** | 23.22 | 0.59 | 82 / 100 | - | 0.235 | 0.937 |
| | **mock (empty sgRNA)** | 25.11 | 0.86 | 38 / 100 | | - | 0.050 |
| | **mock (gfp sgRNA)** | 22.88 | 0.54 | 63 / 100 | | | - |
| # 3 | **WT** | 24.35 | 0.72 | 77 / 100 | - | 0.502 | 1 |
| | **mock (empty sgRNA)** | 26.05 | 1.11 | 28 / 60 | | - | 0.374 |
| | **mock (gfp sgRNA)** | 23.68 | 0.88 | 56 / 100 | | | - |

**Table S8. (A) Statistics of the Independent DAF-16::GFP Nuclear Accumulation Experiments 1. Figure 3 and S2**

| | WT | | | | N-*daf-2* KO | | | | I-*daf-2* KO | | | |
|---|---|---|---|---|---|---|---|---|---|---|---|---|
| | Head | | Intestine | | Head | | Intestine | | Head | | Intestine | |
| | Nuclear | Cytosolic | Nuclear | Cytosolic | Nuclear | Cytosolic | Nuclear | Cytosolic | Nuclear | Cytosolic | Nuclear | Cytosolic |
| **Exp. 1** | 0 | 51 | 0 | 51 | 36 | 8 | 41 | 4 | 2 | 8 | 8 | 2 |
| **Exp. 2** | 0 | 31 | 0 | 59 | 40 | 6 | 66 | 5 | 16 | 16 | 47 | 3 |
| **Exp. 3** | 0 | 49 | 0 | 98 | 43 | 6 | 80 | 8 | 14 | 17 | 41 | 5 |
| **Exp. 4** | 8 | 27 | 2 | 59 | 21 | 0 | 26 | 3 | 15 | 8 | 33 | 2 |

**Table S8. (B) Statistics of the Independent DAF-16::GFP Nuclear Accumulation Experiments 1. Figure 3 and S2**

Head

| ANOVA summary | |
|---|---|
| **F** | 38.49 |
| **P value** | <0.0001 |
| **P value summary** | **** |
| **Significant diff. among means (P < 0.05)?** | Yes |
| **R square** | 0.8953 |

| Tukey's multiple comparisons test | Mean Diff. | 95.00% CI of diff. | Significant? | Summary | Adjusted P Value |
|---|---|---|---|---|---|
| **WT vs. N-*daf-2* KO** | -83.42 | -110.0 to -56.86 | Yes | **** | <0.0001 |
| **WT vs. I-*daf-2* KO** | -39.38 | -65.94 to -12.82 | Yes | ** | 0.0064 |
| **N-*daf-2* KO vs. I-*daf-2* KO** | 44.04 | 17.48 to 70.60 | Yes | ** | 0.0032 |

Body

| ANOVA summary | |
|---|---|
| **F** | 686.6 |
| **P value** | <0.0001 |
| **P value summary** | **** |
| **Significant diff. among means (P < 0.05)?** | Yes |
| **R square** | 0.9935 |

| Tukey's multiple comparisons test | Mean Diff. | 95.00% CI of diff. | Significant? | Summary | Adjusted P Value |
|---|---|---|---|---|---|
| **WT vs. N-*daf-2* KO** | -90.74 | -98.56 to -82.92 | Yes | **** | <0.0001 |
| **WT vs. I-*daf-2* KO** | -88.94 | -96.75 to -81.12 | Yes | **** | <0.0001 |
| **N-*daf-2* KO vs. I-*daf-2* KO** | 1.804 | -6.013 to 9.621 | No | ns | 0.8 |

**Table S9. Statistics of the Independent DAF-16::GFP Nuclear Accumulation Experiments 2. Related to Figure 3**

**ANOVA summary**

| | |
|---|---|
| **F** | 280.6 |
| **P value** | <0.0001 |
| **P value summary** | **** |
| **Significant diff. among means (P < 0.05)?** | Yes |
| **R squared** | 0.9791 |

| **Dunnett's multiple comparisons test** | **Mean Diff.** | **95.00% CI of diff.** | **Significant?** | **Summary** | **Adjusted P Value** | |
|---|---|---|---|---|---|---|
| **Wild-type vs. N-*daf-2* KO** | -7.36 | -8.193 to -6.527 | Yes | **** | <0.0001 | vs N-*daf-2* KO |
| **Wild-type vs. I-*daf-2* KO** | -1.24 | -2.073 to -0.4075 | Yes | ** | 0.0054 | vs I-*daf-2* KO |

| | **Wild-type** | **N-daf-2 KO** | **I-daf-2 KO** |
|---|---|---|---|
| **Exp. #1** | 0 | 5.9 | 0.9 |
| **Exp. #2** | 0.2 | 7.7 | 1.4 |
| **Exp. #3** | 0 | 7.5 | 1.4 |
| **Exp. #4** | 0 | 8 | 1.5 |
| **Exp. #5** | 0.1 | 8 | 1.3 |

**Table S10. Statistics of the Independent Lifespan Experiments 5. Related to Figure 4**

| Trial | Strain | Mean lifespan (Days) | Std. error | Worms (n) | Bonferroni P-value | | | |
|---|---|---|---|---|---|---|---|---|
| | | | | | vs intestine ctrl RNAi | vs intestine *daf-16* RNAi | vs neuronal *daf-2* KO; intestine ctrl RNAi | vs neuronal *daf-2* KO; intestine *daf-16* RNAi |
| # 1 | **Intestine ctrl RNAi** | 17.68 | 0.2 | 37 / 80 | - | 0.0001 | <0.0001 | 0.0009 |
| | **Intestine *daf-16* RNAi** | 15.73 | 0.27 | 55 / 80 | | - | <0.0001 | <0.0001 |
| | **Neuronal *daf-2* KO; intestine ctrl RNAi** | 24.41 | 1.11 | 32 / 80 | | | - | 0.0001 |
| | **Neuronal *daf-2* KO; intestine *daf-16* RNAi** | 19.79 | 0.51 | 43 / 80 | | | | - |
| # 2 | **intestine ctrl RNAi** | 18.3 | 0.31 | 57 / 100 | - | 0.0007 | <0.0001 | 0.0888 |
| | **intestine *daf-16* RNAi** | 16.33 | 0.37 | 25 / 100 | | - | <0.0001 | <0.0001 |
| | **Neuronal *daf-2* KO; intestine ctrl RNAi** | 29.11 | 1.03 | 47 / 100 | | | - | <0.0001 |
| | **Neuronal *daf-2* KO; intestine *daf-16* RNAi** | 19.39 | 0.61 | 53 / 100 | | | | - |
| # 3 | **intestine ctrl RNAi** | 18.74 | 0.29 | 65 /100 | - | 0.0001 | <0.0001 | 0.0303 |
| | **intestine *daf-16* RNAi** | 16.96 | 0.27 | 64 /100 | | - | <0.0001 | <0.0001 |
| | **Neuronal *daf-2* KO; intestine ctrl RNAi** | 28.32 | 0.99 | 56 /100 | | | - | <0.0001 |
| | **Neuronal *daf-2* KO; intestine *daf-16* RNAi** | 21.09 | 0.78 | 72 /100 | | | | - |

| Trial | Strain | Mean lifespan (Days) | Std. error | Worms (n) | Bonferroni P-value | | | |
|---|---|---|---|---|---|---|---|---|
| | | | | | vs neuron ctrl RNAi | vs neuron *daf-16* RNAi | vs intestine *daf-2* KO; neuronctrl RNAi | vs intestine *daf-2* KO; neuron *daf-16* RNAi |
| # 1 | **Neuron ctrl RNAi** | 18.5 | 0.46 | 53 / 80 | - | <0.0001 | <0.0001 | 0.0002 |
| | **Neuron *daf-16* RNAi** | 14.78 | 0.27 | 56 / 80 | | - | <0.0001 | 0.0628 |
| | **Intestin *daf-2* KO; neuron ctrl RNAi** | 30.05 | 1.02 | 49 / 79 | | | - | <0.0001 |
| | **Intestine *daf-2* KO; neuron *daf-16* RNAi** | 15.88 | 0.42 | 54 / 77 | | | | - |
| # 2 | **Neuron ctrl RNAi** | 17.76 | 0.31 | 38 / 81 | - | 0 | <0.0001 | 8.00E-04 |
| | **Neuron *daf-16* RNAi** | 14.74 | 0.37 | 56 / 80 | | - | <0.0001 | 0.1849 |
| | **Intestin *daf-2* KO; neuron ctrl RNAi** | 30.11 | 1.03 | 53 / 81 | | | - | <0.0001 |
| | **Intestine *daf-2* KO; neuron *daf-16* RNAi** | 15.47 | 0.61 | 58 / 80 | | | | - |

**Table S11. Statistics of the Independent Reproductive Span Experiments 2. Related to Figure S3**

| Trial | Strain | Mean reproductive span (Days) | Std. error | Worms (n) | Bonferroni P-value | | | | | |
|---|---|---|---|---|---|---|---|---|---|---|
| | | | | | vs intestine ctrl RNAi | vs intestine *daf-2* RNAi | vs intestine *daf-16* RNAi | vs neuronal *daf-2* KO; intestine ctrl RNAi | vs neuronal *daf-2* KO; intestine *daf-2* RNAi | vs neuronal *daf-2* KO; intestine *daf-16* RNAi |
| # 1 | **intestine ctrl RNAi** | 3.47 | 0.12 | 27/28 | - | 0.0015 | 1 | <0.0001 | <0.0001 | 1 |
| | **intestine *daf-2* RNAi** | 4.2 | 0.13 | 26/28 | | - | 0.0006 | 0.7126 | 0.0151 | 0.1135 |
| | **intestine *daf-16* RNAi** | 3.38 | 0.13 | 28/30 | | | - | <0.0001 | <0.0001 | 1 |
| | **Neuronal *daf-2* KO; intestine ctrl RNAi** | 4.5 | 0.16 | 34/37 | | | | - | 0.3223 | 0.0031 |
| | **Neuronal *daf-2* KO; intestine *daf-2* RNAi** | 4.95 | 0.19 | 36/40 | | | | | - | <0.0001 |
| | **Neuronal *daf-2* KO; intestine *daf-16* RNAi** | 3.6 | 0.18 | 35/41 | | | | | | - |
| # 2 | **intestine ctrl RNAi** | 3.5 | 0.13 | 27/30 | - | | 1 | <0.0001 | | 0.5807 |
| | **intestine *daf-16* RNAi** | 3.43 | 0.14 | 28/30 | | | - | <0.0001 | | 0.3975 |
| | **Neuronal *daf-2* KO; intestine ctrl RNAi** | 4.48 | 0.17 | 35/51 | | | | - | | 0.0484 |
| | **Neuronal *daf-2* KO; intestine *daf-16* RNAi** | 3.85 | 0.19 | 35/50 | | | | | | - |

**Table S12. (A) Statistics of the Brood Size of the Individuals. Related to Figure S3**

| VP303 / Ctrl RNAi | N-*daf-2* KO;VP303 / Ctrl RNAi | VP303 / *daf-16* RNAi | N-*daf-2* KO;VP303 / *daf-16* RNAi |
|---|---|---|---|
| 231 | 291 | 181 | 201 |
| 266 | 260 | 192 | 243 |
| 181 | 273 | 282 | 241 |
| 176 | 257 | 173 | 256 |
| 222 | 257 | 278 | 334 |
| 209 | 233 | 197 | 178 |
| 197 | 200 | 244 | 293 |
| 238 | 180 | 184 | 225 |
| 210 | 235 | 263 | 221 |
| 203 | 253 | 203 | 227 |
| 246 | 267 | 241 | 271 |
| 123 | 133 | 208 | 179 |
| 207 | 261 | 224 | 205 |
| 234 | 318 | 269 | 251 |
| 309 | 239 | 240 | 240 |
| 251 | 200 | 228 | 196 |
| 200 | 266 | 231 | 159 |
| 138 | 255 | 214 | 190 |
| 258 | 241 | 213 | 271 |
| 209 | 193 | 181 | 206 |
| 157 | 229 | 235 | 258 |
| 204 | 221 | 117 | 202 |
| 223 | 266 | 244 | 234 |
| 218 | 224 | 201 | 155 |
| | 177 | 221 | 215 |
| | 230 | | 127 |
| | 241 | | 195 |
| | | | 206 |
| | | | 191 |
| | | | 255 |
| | | | 169 |
| | | | 233 |

**Table S12. (B) Statistics of the Brood Size of the Individuals. Related to Figure 3**

| ANOVA summary | |
|---|---|
| **F** | 1.775 |
| **P value** | 0.1566 |
| **P value summary** | ns |
| **Significant diff. among means (P < 0.05)?** | No |
| **R square** | 0.0487 |

| Tukey's multiple comparisons test | Mean Diff. | 95.00% CI of diff. | Significant? | Summary | Adjusted P Value |
|---|---|---|---|---|---|
| **VP303 / Ctrl RNAi vs. N-*daf-2* KO;VP303 / Ctrl RNAi** | -24.12 | -53.49 to 5.253 | No | ns | 0.1462 |
| **VP303 / Ctrl RNAi vs. VP303 / *daf-16* RNAi** | -5.643 | -35.56 to 24.28 | No | ns | 0.9606 |
| **VP303 / Ctrl RNAi vs. N-*daf-2* KO;VP303 / *daf-16* RNAi** | -6.677 | -34.95 to 21.60 | No | ns | 0.9266 |
| **N-*daf-2* KO;VP303 / Ctrl RNAi vs. VP303 / *daf-16* RNAi** | 18.48 | -10.58 to 47.54 | No | ns | 0.35 |
| **N-*daf-2* KO;VP303 / Ctrl RNAi vs. N-*daf-2* KO;VP303 / *daf-16* RNAi** | 17.44 | -9.917 to 44.80 | No | ns | 0.3476 |
| **VP303 / *daf-16* RNAi vs. N-*daf-2* KO;VP303 / *daf-16* RNAi** | -1.034 | -28.98 to 26.91 | No | ns | 0.9997 |

**Table S13. (A) Statistics of the Independent DAF-16::GFP Nuclear Accumulation Experiments 2. Related to Figure 5 and S4**

**Ctrl RNAi**

| WT | | | | N-*daf-2* KO | | | | I-*daf-2* KO | | | |
|---|---|---|---|---|---|---|---|---|---|---|---|
| Head | | Intestine | | Head | | Intestine | | Head | | Intestine | |
| Nuclear | Cytosolic | Nuclear | Cytosolic | Nuclear | Cytosolic | Nuclear | Cytosolic | Nuclear | Cytosolic | Nuclear | Cytosolic |
| 1 | 16 | 1 | 19 | 15 | 1 | 19 | 1 | 7 | 7 | 15 | 0 |
| 0 | 34 | 0 | 58 | 17 | 5 | 22 | 4 | 8 | 8 | 20 | 0 |
| 0 | 23 | 0 | 33 | 23 | 5 | 31 | 3 | 6 | 10 | 21 | 0 |
| 0 | 29 | 0 | 45 | 37 | 9 | 66 | 7 | 13 | 14 | 28 | 4 |

***daf-18* RNAi**

| WT | | | | N-*daf-2* KO | | | | I-*daf-2* KO | | | |
|---|---|---|---|---|---|---|---|---|---|---|---|
| Head | | Intestine | | Head | | Intestine | | Head | | Intestine | |
| Nuclear | Cytosolic | Nuclear | Cytosolic | Nuclear | Cytosolic | Nuclear | Cytosolic | Nuclear | Cytosolic | Nuclear | Cytosolic |
| 0 | 26 | 0 | 49 | 19 | 1 | 13 | 7 | 4 | 6 | 7 | 3 |
| 0 | 32 | 0 | 48 | 5 | 6 | 6 | 10 | 4 | 7 | 11 | 2 |
| 0 | 31 | 0 | 52 | 21 | 10 | 32 | 18 | 7 | 12 | 19 | 5 |
| 0 | 26 | 0 | 52 | 21 | 18 | 42 | 17 | 12 | 20 | 39 | 12 |

**Table S13. (B) Statistics of the Independent DAF-16::GFP Nuclear Accumulation Experiments 2. Related to Figure 5 and S4**

| | ANOVA table | SS | DF | MS | F (DFn, DFd) | P value |
|---|---|---|---|---|---|---|
| **Head** | **Interaction** | 270.8 | 2 | 135.4 | F (2, 18) = 1.425 | P=0.2663 |
| | **KO** | 21844 | 2 | 10922 | F (2, 18) = 115.0 | P<0.0001 |
| | **RNAi** | 526.2 | 1 | 526.2 | F (1, 18) = 5.540 | P=0.0302 |
| | **Residual** | 1710 | 18 | 95 | | |
| | **Sidak's multiple comparisons tes** | **Mean Diff.** | **95.00% CI of diff.** | **Significant?** | **Summary** | **Adjusted P Value** |
| | | | | | | |
| | **Ctrl RNAi - *daf-18* RNAi** | | | | | |
| | **WT** | 1.471 | -16.66 to 19.60 | No | ns | 0.9954 |
| | **N-*daf-2* KO** | 17.89 | -0.2437 to 36.02 | No | ns | 0.0538 |
| | **I-*daf-2* KO** | 8.736 | -9.398 to 26.87 | No | ns | 0.5275 |

| | ANOVA table | SS | DF | MS | F (DFn, DFd) | P value |
|---|---|---|---|---|---|---|
| **Body** | **Interaction** | 576.4 | 2 | 288.2 | F (2, 18) = 4.836 | P=0.0209 |
| | **KO** | 34024 | 2 | 17012 | F (2, 18) = 285.5 | P<0.0001 |
| | **RNAi** | 1323 | 1 | 1323 | F (1, 18) = 22.19 | P=0.0002 |
| | **Residual** | 1073 | 18 | 59.6 | | |
| | **Sidak's multiple comparisons tes** | **Mean Diff.** | **95.00% CI of diff.** | **Significant?** | **Summary** | **Adjusted P Value** |
| | | | | | | |
| | **Ctrl RNAi - *daf-18* RNAi** | | | | | |
| | **WT** | 1.25 | -13.11 to 15.61 | No | ns | 0.9943 |
| | **N-*daf-2* KO** | 23.98 | 9.616 to 38.34 | Yes | ** | 0.0011 |
| | **I-*daf-2* KO** | 19.31 | 4.949 to 33.67 | Yes | ** | 0.007 |

**Table S14. Strain List. Related to STAR Method**

| Strain | Genotype | Comment |
|---|---|---|
| N2 | | Wild type |
| NL2098 | *rrff-1(pk1417) I* | Germline specific RNAi |
| NR222 | *rde-1(ne219) V* (outcrossed 3x)*; kzIs9[lin26p::nls::gfp + lin-26p::rde-1 + rol-6(su1006)]* | Hypodormis specific RNAi |
| NR350 | *rde-1(ne219) V* (outcrossed 1x)*; kzIs20[hlh-1p::rde-1 + sur-5p::nls::gfp]* | Muscle specific RNAi |
| TU3401 | *sid-1(pk3321) V* (outcrossed 0x)*; uIs69[myo-2p::mCherry + unc-119p::sid-1]* | Neuron specific RNAi |
| VP303 | *rde-1(ne219) V* (outcrossed 3x)*; kbIs7[nhx-2p::rde-1; rol-6(su1006)]* | Intestine specific RNAi |
| KN259 | *huIs33[sod-3::gfp + rol-6(su1006)]* | |
| TJ356 | *zIs356[daf-16::gfp + rol-6(su1006)]* | |
| FJ224 | *unc-31(e298) Ⅳ; dpy-11(e224) Ⅴ* (outcrossed 3x) | |
| DCL569 | *mkcSi13 [sun-1p::rde-1::sun-1 3′ UTR + unc-119(+)] II; rde-1(ne219) V* | Germline specific RNAi |
| NIS1700 | *kyEx1700[myo-2p::mCherry + U6p::sgRNA(empty) + eft-3p::Cas9 + dpy-30p::wGxxFP(empty)]* | N2 mock #1 |
| NIS1701 | *kyEx1701[myo-2p::mCherry + U6p::gfp sgRNA + eft-3p::Cas9 + dpy-30p::wGxxFP(empty)]* | N2 mock #2 |
| NIS1702 | *kyEx1702[myo-2p::mCherry + U6p::daf-2 sgRNA + dpy-5p::Cas9 + dpy-30p::wGxxFP(daf-2)]* | hypodormis specific KO |
| NIS1703 | *kyEx1703[myo-2p::mCherry + U6p::daf-2 sgRNA + unc-54p::Cas9 + dpy-30p::wGxxFP(daf-2)]* | muscle specific KO |
| NIS1704 | *kyEx1704[myo-2p::mCherry + U6p::daf-2 sgRNA + rgef-1p::Cas9 + dpy-30p::wGxxFP(daf-2)]* | neuron specific KO |
| NIS1705 | *kyEx1705[myo-2p::mCherry + U6p::daf-2 sgRNA + gly-19p::Cas9 + dpy-30p::wGxxFP(daf-2)]* | intestine specific KO |
| NIS1706 | *kyEx1706[myo-2p::mCherry + U6p::daf-2 sgRNA + unc-54p::Cas9 + dpy-30p::wGxxFP(daf-2)]* | |
| NIS1707 | *kyEx1707[myo-2p::mCherry + U6p::daf-2 sgRNA + ehn-3p::Cas9 + dpy-30p::wGxxFP(daf-2)]* | |
| NIS1708 | *unc-31(e298) Ⅴ (outcrossed 3x); kyEx1704[myo-2p::mCherry + U6p::daf-2 sgRNA + rgef-1p::Cas9 + dpy-30p::wGxxFP(daf-2)]* | |
| NIS1709 | *unc-31(e298) Ⅴ (outcrossed 3x); kyEx1705[myo-2p::mCherry + U6p::daf-2 sgRNA + gly-19p::Cas9 + dpy-30p::wGxxFP(daf-2)]* | |
| NIS1710 | *huIs33[sod-3::gfp + rol-6(su1006)]; kyEx1704[myo-2p::mCherry + U6p::daf-2 sgRNA + rgef-1p::Cas9]* | |
| NIS1711 | *huIs33[sod-3::gfp + rol-6(su1006)]; kyEx1705[myo-2p::mCherry + U6p::daf-2 sgRNA + gly-19p::Cas9]* | |
| NIS1712 | *huIs33[sod-3::gfp + rol-6(su1006)]; kyEx1702[myo-2p::mCherry + U6p::daf-2 sgRNA + dpy-5p::Cas9]* | |
| NIS1713 | *zIs356[daf-16::gfp + rol-6(su1006)]; kyEx1704[myo-2p::mCherry + U6p::daf-2 sgRNA + rgef-1p::Cas9]* | |
| NIS1714 | *zIs356[daf-16::gfp + rol-6(su1006)]; kyEx1705[myo-2p::mCherry + U6p::daf-2 sgRNA + gly-19p::Cas9]* | |
| NIS1715 | *huIs33[sod-3::gfp + rol-6(su1006)]; kyEx1708[myo-2p::mCherry + eft-3p::Cas9]* | sod-3::gfp mock |
| NIS1716 | *rde-1(ne219) V* (outcrossed 3x)*; kbIs7[nhx-2p::rde-1; rol-6(su1006)]; kyEx1705[myo-2p::mCherry + U6p::daf-2 sgRNA + rgef-1p::Cas9]* | neuron specific KO and intestine specific RNAi |
| NIS1717 | *sid-1(pk3321) V* (outcrossed 0x)*; uIs69[myo-2p::mCherry + unc-119p::sid-1]; kyEx1706[myo-3p::mCherry + U6p::daf-2 sgRNA + gly-19p::Cas9]* | intesitine specific KO and neuron specific RNAi |
| NIS1718 | *huIs33[sod-3::gfp + rol-6(su1006)]; kyIs1705[myo-2p::mCherry + U6p::daf-2 sgRNA + rgef-1p::Cas9]* (outcrossed 0x) *#1* | |
| NIS1719 | *huIs33[sod-3::gfp + rol-6(su1006)]; kyIs1705[myo-2p::mCherry + U6p::daf-2 sgRNA + rgef-1p::Cas9]* (outcrossed 0x) *#2* | |
| NIS1720 | *huIs33[sod-3::gfp + rol-6(su1006)]; kyIs1706[myo-2p::mCherry + U6p::daf-2 sgRNA + gly-19p::Cas9]* (outcrossed 0x) *#1* | |
| NIS1721 | *huIs33[sod-3::gfp + rol-6(su1006)]; kyIs1706[myo-2p::mCherry + U6p::daf-2 sgRNA + gly-19p::Cas9]* (outcrossed 0x) *#2* | |
| NIS1722 | *huIs33[sod-3::gfp + rol-6(su1006)]; kyIs1706[myo-2p::mCherry + U6p::daf-2 sgRNA + gly-19p::Cas9]* (outcrossed 0x) *#3* | |
| NIS1723 | *zIs356[daf-16::gfp + rol-6+C22:C40(su1006)]; kyIs1705[myo-2p::mCherry + U6p::daf-2 sgRNA + rgef-1p::Cas9] (outcrossed 0x)* | |
| NIS1724 | *sid-1(qt9) Ⅴ* (outcrossed x1); *huIs33[sod-3::gfp + rol-6(su1006)]; kyIs1705[myo-2p::mCherry + U6p::daf-2 sgRNA + rgef-1p::Cas9]* (outcrossed 2x) | |
| NIS1725 | *unc-31(e298) Ⅳ* (outcrossed 5x) | |
| NIS1726 | *unc-31(e298) Ⅳ* (outcrossed 6x); *huIs33[sod-3::gfp + rol-6(su1006)]* | |
| NIS1727 | *unc-31(e298) Ⅳ* (outcrossed 6x); *huIs33[sod-3::gfp + rol-6(su1006)]; kyIs1705[myo-2p::mCherry + U6p::daf-2 sgRNA + rgef-1p::Cas9]* (outcrossed 2x) | |
| NIS1728 | *unc-64(e246) Ⅲ* (outcrossed 1x); *huIs33[sod-3::gfp + rol-6(su1006)]* | |
| NIS1729 | *unc-64(e246) Ⅲ* (outcrossed 1x); *huIs33[sod-3::gfp + rol-6(su1006)]; kyIs1705[myo-2p::mCherry + U6p::daf-2 sgRNA + rgef-1p::Cas9]* (outcrossed 2x) | |
| NIS1730 | *unc-64(e246) Ⅲ* (outcrossed 1x); *huIs33[sod-3::gfp + rol-6(su1006)]; kyIs1706[myo-2p::mCherry + U6p::daf-2 sgRNA + gly-19p::Cas9]* (outcrossed 2x) | |

**Table S15. Primer List. Related to STAR Method**

| Primer | Sequence |
|---|---|
| ***daf-2*** **RNAi Fw** | 5'-ATCCCTCCGATCAATGCGAC-3' |
| ***daf-2*** **RNAi Rv** | 5'-TGCTGTTGGAGAGCTGCTGC-3' |
| ***daf-16*** **RNAi Fw** | 5'-AGAATGAAGGAGCGGAAAG-3' |
| ***daf-16*** **RNAi Rv** | 5'-ACTGTTCAACTCGTGGTATG-3' |
| **pDD162_non-sgRNA Fw** | 5'-AAGTGGCACCGAGTCGGTGCACACCTTAAAGGCGCA-3' |
| **pDD162_non-sgRNA Rv** | 5'-GCACCGACTCGGTGCCTGCGCCTTTAAGGTGTACAG-3' |
| **empty sgRNA Fw** | 5'-GGGAGACCGGCAGATACTTTATTGTGCCGCCAAGA-3' |
| **empty sgRNA Rv** | 5'-TATCGATAAGCTTGAAGTGCACCATATGCGGTGTG-3' |
| ***daf-2*** **sg RNA Fw** | 5'-CGGACAACAGTGTGGAGGTTTTAGAGCTAGAAATAGC-3' |
| ***daf-2 sg RNA*** **Rv** | 5'-CACACTGTTGTCCGAGCCAAGAACATCTCGCAATAGGA-3' |
| **P*rgef-1*** **Fw** | 5'-ATGATTACGCCAAGCGATAAACCGTTTGGATGAGC-3' |
| **P*rgef-1*** **Rv** | 5'-GTATTTTTTGTCCATCGTCGTCGTCGTCGATGCCG-3' |
| **P*gly-19*** **Fw** | 5'-ATGATTACGCCAAGCCAAATATTCTCATTTCAAAA-3' |
| **P*gly-19*** **Rv** | 5'-GTATTTTTTGTCCATCTGGAAATTTAAATTTAATT-3' |
| **P*dpy-5*** **Fw** | 5'-CATGATTACGCCAAGCCATAGAAGTATGGGCCAAAA-3' |
| **P*dpy-5*** **Rv** | 5'-GTATTTTTTGTCCATTTTCTGAAAACACAGAGACT-3' |
| **P*ehn-3*** **Fw** | 5'-ATGATTACGCCAAGCTAATCTAGAAAAATACGACA-3' |
| **P*ehn-3*** **Rv** | 5'-GTATTTTTTGTCCATTTTGTAATTTGGAAGCTGGG-3' |
| **wGxxFP insert Fw** | 5'-CCGCTGCAGTCGACAGATCTTGCACTACTGGAAAACTACC-3' |
| **wGxxFP insert Rv** | 5'-CAATTGGAGTATTTTGTTGA-3' |
| **wGxxFP vector Fw** | 5'-AAAATACTCCAATTGGCGAT-3' |
| **wGxxFP vector Rv** | 5'-TGTCGACTGCAGCGGCCGCGTTGATAATGGTCTGCTAGT-3' |
| **wGxxFP (*daf-2*) Fw** | 5'-ATTATCAACGCGGCCATGACGCCAGGCTTCTTCAC-3' |
| **wGxxFP (*daf-2*) Rv** | 5'-CAGTAGTGCAAGATCGAGATAGTCACGGAGATTTC-3' |
